# EnergiQ: A Prescriptive Large Language Model-Driven Intelligent Platform for Interpreting Appliance Energy Consumption Patterns

**DOI:** 10.3390/s25164911

**Published:** 2025-08-08

**Authors:** Christoforos Papaioannou, Ioannis Tzitzios, Alexios Papaioannou, Asimina Dimara, Christos-Nikolaos Anagnostopoulos, Stelios Krinidis

**Affiliations:** 1 Management Science and Technology Department, Democritus University of Thrace, 65404 Kavala, Greece; tzijonnis@gmail.com (I.T.); alpapaio@mst.duth.gr (A.P.); adimara@affil.duth.gr (A.D.); krinidis@mst.duth.gr (S.K.); 2 Department of Cultural Technology and Communication, Intelligent Systems Lab, University of the Aegean, 81100 Mytilene, Greece

**Keywords:** smart sensors, IoT-based energy monitoring, appliance-level energy profiling, large language models, anomaly detection, human-centric energy feedback

## Abstract

The increased usage of smart sensors has introduced both opportunities and complexities in managing residential energy consumption. Despite advancements in sensor data analytics and machine learning (ML), existing energy management systems (EMS) remain limited in interpretability, adaptability, and user engagement. This paper presents EnergiQ, an intelligent, end-to-end platform that leverages sensors and Large Language Models (LLMs) to bridge the gap between technical energy analytics and user comprehension. EnergiQ integrates smart plug-based IoT sensing, time-series ML for device profiling and anomaly detection, and an LLM reasoning layer to deliver personalized, natural language feedback. The system employs statistical feature-based XGBoost classifiers for appliance identification and hybrid CNN-LSTM autoencoders for anomaly detection. Through dynamic user feedback loops and instruction-tuned LLMs, EnergiQ generates context-aware, actionable recommendations that enhance energy efficiency and device management. Evaluations demonstrate high appliance classification accuracy (94%) using statistical feature-based XGBoost and effective anomaly detection across varied devices via a CNN-LSTM autoencoder. The LLM layer, instruction-tuned on a domain-specific dataset, achieved over 91% agreement with expert-written energy-saving recommendations in simulated feedback scenarios. By translating complex consumption data into intuitive insights, EnergiQ empowers consumers to engage with energy use more proactively, fostering sustainability and smarter home practices.

## 1. Introduction

In contemporary homes, the spread of electrical appliances has also posed a huge challenge for consumers in controlling and assessing their power usage efficiently. Every appliance, be it a refrigerator, washing machine, air conditioner, or oven, has its own distinct functions, working modes, and power utilization patterns. These features can differ enormously, not just among different brands but even among various models of a single brand, leading to differences in the way appliances function and consume energy [1]. This complicates how consumers can grasp the behavior of their appliances or make comparisons based on efficiency. The difficulty in monitoring consumption, detecting inefficiencies, or making intelligent usage decisions is due to a lack of standardization and transparency. Moreover, the technical nature of translating technical specifications and energy information is daunting to the non-expert consumer, making the adoption of cleaner and economically efficient energy cultures impossible [2].

Recent advances in smart home and Energy Management System (EMS) technologies have been driven primarily by innovations in Machine Learning (ML) algorithms, sensor fusion, and smart metering. Current practices leverage high-resolution data, clustering techniques, and consumption pattern analytics to support appliance scheduling and energy usage optimization. ML methods such as K-means clustering and Support Vector Machines (SVMs) are commonly employed to identify appliance load profiles. In parallel, privacy-preserving mechanisms, such as homomorphic encryption and the SAFE approach, are increasingly adopted to ensure user data protection during appliance-level monitoring and scheduling. Even with such developments, some areas of research are still lacking. Some of them include few real-world applications, and a few are studies on the impact of long-term behavioral effects on users. There is also the issue of convergence between the IoT and smart grid platforms, bringing pre-existing scalability and interoperability challenges [3]. This aligns with hierarchical resource management approaches in smart cities, where structured IoT frameworks have been proposed to improve system scalability and decision-making efficiency [4].

The importance of robust appliance load monitoring infrastructure is also being realized increasingly, and an investigation of the Internet of Behavior (IoB) needs to be performed further in order to better enhance EMS [5]. One emerging line of research is a reverse paradig- titled energy profiling, where patterns of electricity usage are derived from appliance use history rather than power signals. This methodology has disclosed profound vulnerabilities within existing practice, including too broadly defined appliance categories and poorly framed prediction models. Combined, these findings suggest the need for independent, scalable, and intelligent oversight engineering management system designs that synthesize behavioral science and support real-time decision-making [6].

The intersection of Large Language Models (LLMs) and machine learning (ML) has demonstrated revolutionary capabilities in various industries, particularly the energy sector. Self-supervised learning and Graph Neural Networks (GNNs) have been instrumental in modeling complex energy systems, such as renewable integration and power grid management. Recent studies provide useful guidance on the deployment of LLM in NLP tasks, such as the need to know the structure of the model and performance metrics to facilitate successful implementation in real-world energy applications [7]. A new integration between Graph Machine Learning (Graph ML) and Large Language Models (LLMs) promotes graph-based modeling, which allows optimal energy distribution and strategic resource allocation [8]. LLMs have been used in the power industry to control unstructured data by responding to technical queries and aggregating grid data, both necessary in controlling complex systems in transitioning towards renewable sources.

Recent innovations underscore the growing integration of Large Language Models in enhancing energy system performance. Emerging research directions include the optimization of domain-specific data infrastructures, integration of energy-focused tools, and application of LLMs in safety-critical environments [9]. Advances in transformer-based LLM architectures have enabled more efficient data processing and contextual reasoning, even with limited datasets. However, despite their widespread adoption in various energy domains, such as grid management and predictive maintenance, the application of LLMs at the appliance level remains largely unexplored. This area represents a significant untapped opportunity for advancing home Energy Management Systems (EMSs) by providing intelligent, personalized consumption feedback and fostering user-centric energy optimization [10].

Within this context, this paper presents EnergiQ, a smart, LLM-driven platform that is coded to interpret appliance-level energy usage patterns and overcome persistent shortcomings in existing EMS’s and smart home infrastructures. EnergiQ is designed as an end-to-end solution that combines sophisticated machine learning algorithms, real-time sensor data streams, and the large-scale interpretive power of LLMs to deliver a seamless and intelligent energy-monitoring experience. The platform not only offers users extensive information on the performance and efficiency of specific household appliances, but also personalized recommendations on home appliance level, anomaly alerts, and tailored guidance, all communicated in natural language that non-expert users can easily understand. By integrating fine-grained data collection via smart plugs and combining edge processing with central reasoning engines, EnergiQ effectively bridges the gap between technical energy metrics and end-user understanding. Its scalable and modular design allows it to be flexible in various residential environments, and its focus on user interaction and behavior-based feedback loops is a move towards more intuitive and proactive EMS design. By centering on user interaction and adaptive feedback, EnergiQ promotes proactive engagement with energy use, ultimately fostering more sustainable, efficient, and informed household energy practices. The main novelties of this paper are as follows:

- A prescriptive, LLM-driven platform, EnergiQ, has been developed to improve household energy awareness through interpretable, appliance-specific recommendations.
- A scalable, modular architecture has been proposed, leveraging edge computing and plug-and-play IoT devices to enable straightforward deployment across diverse residential environments.
- A time-series anomaly detection pipeline and feedback loop have been implemented to adapt over time to user behavior, supporting personalized energy efficiency strategies.

The rest of this paper is organized as follows: Section 2 provides an overview of the literature on energy consumption analysis and intelligent systems, positioning our contribution in the larger research context. Section 3 presents the expected EnergiQ framework, outlining its architecture, data pipelines, and integration of large language models for appliance-level energy interpretation. This is followed by Section 4, where the platform implementation and example use cases illustrating the practical usefulness of EnergiQ are presented. Section 5 and Section 6 present experimental results and use case scenarios that demonstrate how end-users interact with and benefit from the platform’s capabilities. Lastly, Section 7 concludes the paper by summarizing the main results and positing lines for future work.

## 2. Related Work

### 2.1. Machine Learning Techniques

Non-Intrusive Load Monitoring (NILM), also known as energy disaggregation, estimates appliance-level energy consumption using only the total household or building power usage, eliminating the need for dedicated sensors on each appliance. Over the past three decades, NILM has advanced significantly, driven by the proliferation of smart meters and the integration of machine learning techniques that enable more accurate and scalable solutions. Research has explored both low-frequency and high-frequency NILM algorithms, applying signal processing and statistical inference techniques as well as more recent graph-based and deep learning approaches [11]. Another comprehensive review emphasizes the need for standardized performance evaluation and highlights the challenge of comparing results across the growing number of public datasets. It also introduces the Non-Intrusive Load Monitoring Toolkit NILMTK, an open-source benchmarking toolkit that enables reproducible comparisons of NILM approaches [12]. The need to improve the generalization ability of NILM algorithms is also identified as a major challenge, particularly regarding model portability across diverse household settings.

In parallel, various NILM techniques such as Hidden Markov Models (HMMs), k-means clustering, and Gaussian Mixture Models (GMMs) have been proposed, though challenges remain in identifying low-power devices due to low sampling rates [13]. The document also highlights increased integration of NILM with smart grid applications, electric vehicles, and distributed energy resources, as a reflection of its increasing use in new energy systems [13]. Recent advances in predictive modeling have leveraged deep learning architectures such as Long Short-Term Memory (LSTM) and Gated Recurrent Unit (GRU) networks, achieving a strong performance in forecasting appliance-level consumption from time-series data [14]. Similarly, advanced proactive anomaly detection techniques have been explored for multi-pattern home appliances, demonstrating their potential in optimizing household energy consumption [15]. Together, these advancements illustrate how NILM has evolved from a theoretical concept into a data-driven and interdisciplinary field with practical applications in both residential and commercial energy management.

Building on the foundations of NILM, machine learning has become a key approach for identifying energy usage patterns and enhancing load forecasting. Several studies describe a natural progression from classical statistical models, such as ARMA and ARIMA, known for handling both stationary and non-stationary time series, to modern machine learning techniques that better capture the dynamic behavior of power systems. With the rise of smart grids, research has shifted toward algorithms like Support Vector Machines (SVMs) and Bayesian Regularization Backpropagation Neural Networks (BRBNNs), which offer improved predictive accuracy and generalization. However, the growing complexity and volume of energy data have highlighted the limitations of traditional ML approaches, prompting a shift toward deep learning architectures such as Recurrent Neural Networks (RNNs), LSTMs, and GRUs, which are better suited for modeling temporal and sequential patterns [16].

Another prominent research direction explores deep learning for appliance-level monitoring, comparing Intrusive Load Monitoring (ILM) and NILM, with the latter favored for its cost-effectiveness and scalability. These approaches are categorized into data-driven and feature-based methods, reflecting a shift from traditional signal processing techniques to advanced neural architectures like multi-layer LSTMs and multiscale residual networks for precise appliance identification. The method suggested in the paper uses an LSTM-based multi-meter technique to identify a maximum of 16 appliances with competitive performance with the existing state-of-the-art literature [17].

Broader applications of AI and ML in energy systems have been explored, particularly in developing countries. Research highlights the role of machine learning in areas such as short-term renewable forecasting, electricity theft detection, and predictive maintenance [18]. Emerging structures like DimNet have further advanced the understanding and simulation of intricate energy dynamics. Moreover, AI-based socioeconomic modeling assists policymakers in designing fair and sustainable energy models, reinforcing the importance of AI and ML in addressing global energy challenges [18].

The integration of deep learning in NILM has garnered significant attention, particularly with models like Neural-NILM and multiscale residual networks, which outperform traditional methods in disaggregating complex appliances such as washing machines and dishwashers [19]. A review underscores the transition from conventional methods to deep learning, emphasizing the superior performance of these models. It also stresses the need for publicly available standardized datasets for performance measurement and benchmarks of NILM models using metrics like Mean Absolute Error (MAE) and F1-score. Despite progress, challenges in implementing NILM models across varied environments remain, indicating areas for future research [19]. Collectively, these studies illustrate the co-evolution of statistical, machine learning, and deep learning methods in modern energy systems, emphasizing the importance of data-driven approaches not only for forecasting and classification but also for broader challenges in smart grid development and sustainable energy policy.

Recent developments in LLMs have brought along new opportunities for scaling automation, interpretability, and user friendliness to power systems. Among the interesting application areas is that of Building Energy Modeling (BEM), wherein conventional model building methodologies are tiresome and involved in terms of requiring brute manpower and intensive domain expertise. The release of Eplus-LLM, a computer platform based on an extremely optimized LLM, reveals the automatic mapping of natural language descriptions to EnergyPlus simulation models. It makes modeling more than 95% faster with precision, hence, building simulation software is a possibility for all and provides support for large-scale urban energy modeling endeavors [20].

The platform leverages the multimodal and contextual nature of LLMs (e.g., the T5 model) to learn about various types of input, making it resilient to real-user variation like colloquial usage or misspelling. This is promising for smart homes and energy-efficient building design, where seamless automation can be revolutionary.

Beyond applications, LLMs also pose questions regarding the very form of algorithms and explainability. Recent research proposes a formal approach to analyzing and optimizing the design of LLM-based systems instead of heuristic trial-and-error. Treating LLM algorithms as computational graphs and studying patterns such as hierarchical and parallel decomposition, the authors provide approaches to both enhancing efficiency and interpretability in such systems [21]. These results are intriguing for energy applications where reliability, performance tuning, and transparency are valued, e.g., time-series energy forecasting or anomaly detection. Cumulatively, these results showcase not just how LLMs are facilitating the progress of energy modeling and simulation, but also becoming the focal point for the development of interpretable, scalable, and smart energy management solutions.

### 2.2. Wireless Communication Techniques for IoT Sensing

Efficient and reliable wireless communication plays a critical role in IoT-based energy management platforms like EnergiQ, where smart sensors and plugs continuously transmit data for analysis. Recent advances in modulation schemes designed for low-power and robust wireless communications have the potential to significantly enhance IoT device performance.

The multi-carrier initial-condition-index-aided differential chaos shift keying (MC-ICI-DCSK) scheme offers a novel approach for multipath fading channels by encoding information in the initial conditions of chaotic sequences. This technique improves bit error rates, spectral efficiency, and energy efficiency, making it a promising candidate for short-range, low-power IoT communications [22].

Similarly, enhancements in chirp spread spectrum modulation, such as the multiple-slope-keying CSS (MSK-CSS) and multi-layer superposition modulation (MLSM) schemes, have been proposed to increase the data rate of low-power wide-area networks (LPWANs) like LoRa. These schemes provide a flexible trade-off between data throughput and bit error rate performance, which is essential for scalable and reliable IoT deployments [23].

Although EnergiQ primarily focuses on intelligent energy analytics and user interaction leveraging sensor data, integrating such advanced physical-layer communication techniques could improve the system’s overall efficiency by reducing transmission energy, enhancing data reliability, and enabling scalable sensor networks. Exploring these wireless communication innovations presents a promising direction for future work to optimize real-time energy monitoring and device management in smart homes.

### 2.3. Smart Energy Platforms

Traditional platforms in home automation systems like Home Assistant and NRG4-U have been trendsetters in adaptive and personalized home energy management. Home Assistant is an open-source Internet of Things platform that connects several smart devices using a common control interface, with the possibility of extensibility as well as community backing, albeit privacy and security issues owing to the open nature [24]. NRG4-U, however, has an advanced, unobtrusive home energy management system (HEMS) for individual user load profiles based on hybrid machine learning techniques. It constructs custom energy and comfort profiles without historics or preprocessing, hence ideal for mass installations [25].

As compared to this, next-generation platforms are revolutionizing with smart Battery Management Systems (IBMS) and end-edge-cloud architectures. These systems address the shortcomings of traditional BMS through the use of digital twins, IoT, blockchain, and cloud computing to provide real-time diagnosis, predictive maintenance, and secure data management. Closed platforms employing IBMS architectures tend to be proprietary but provide better data integrity and control, which is critical in electric vehicles and energy storage devices. The transition towards multilayer computing (end, edge, and cloud) increases the performance, scalability, and responsiveness of BMS functionality as well as supporting innovative functions such as fault prognosis, SOC/SOH estimation, and thermal management [26].

The technology landscape shows a futuristic overlap of open and closed systems. Open platforms such as Home Assistant with BMS capability can be beneficial for flexibility and community innovation, but need robust security. By contrast, closed IBMS platforms offer reliability and commercial readiness, particularly for mission-critical applications like EVs and grid-scale storage. Selection among them depends on the application: customization and flexibility in smart homes and accuracy, security, and fault tolerance in industrial and automotive use cases. Table 1 summarizes traditional and state-of-the-art platforms of home automation and battery management systems by comparing their most salient features, levels of openness, and corresponding references. All the identified findings regarding current platforms are summarized in Table 1, which provides a structured comparison between traditional and advanced platforms for home automation and battery management systems.

Overall, in comparison to traditional and other machine learning-driven energy management platforms, EnergiQ introduces distinct advancements in both interpretability and adaptability within real-world scenarios. Conventional platforms, such as Home Assistant, provide modular device integration and rudimentary energy insights but lack the fine-grained, appliance-level interpretability and adaptive reasoning layer found in EnergiQ. Similarly, systems like NRG4-U employ machine learning for user profiling but fall short in providing continuous feedback loops and natural language interpretability that empower user action. EnergiQ bridges these gaps by offering a tightly integrated LLM-driven reasoning engine that translates raw energy metrics into context-aware, actionable advice, dynamically adjusted to user behavior. In real-world deployments, EnergiQ demonstrated superior adaptability through its retraining mechanisms based on user feedback, achieving more accurate anomaly detection and personalization over time compared to static, one-size-fits-all solutions. This positions EnergiQ as a novel contribution to the field, combining technical precision with user-centric design to actively shape energy-conscious behavior in households.

Table 2 gives an overall evaluation of the key research gaps and limitations recognized in existing studies on NILM, machine learning, and LLM-based methods for smart energy systems and corresponding solutions put forward in the literature to overcome such challenges.

## 3. Proposed Framework

### 3.1. System Overview and Architecture

EnergiQ is an intelligent, user-friendly platform that enables households to understand, and optimize appliance-level energy consumption through seamless automation and personalized feedback. The user journey begins with the installation of a plug-and-play IoT setup comprising smart plugs and a pre-configured local gateway (e.g., a Raspberry Pi). Once the appliance is powered on, the system will automatically record real-time measurements of power, current, and voltage. The measurements are transmitted securely to the cloud through the MQTT protocol with TLS encryption, and authenticated through using token-based access control mechanisms. This provides confidentiality and data integrity during the data transport. Through the EnergiQ web or mobile interface, users are guided to easily pair each smart plug with the corresponding appliance using intuitive menus. This process takes just a few seconds and requires no technical expertise, enabling accurate device-level tracking with minimal effort.

**Figure 1:**
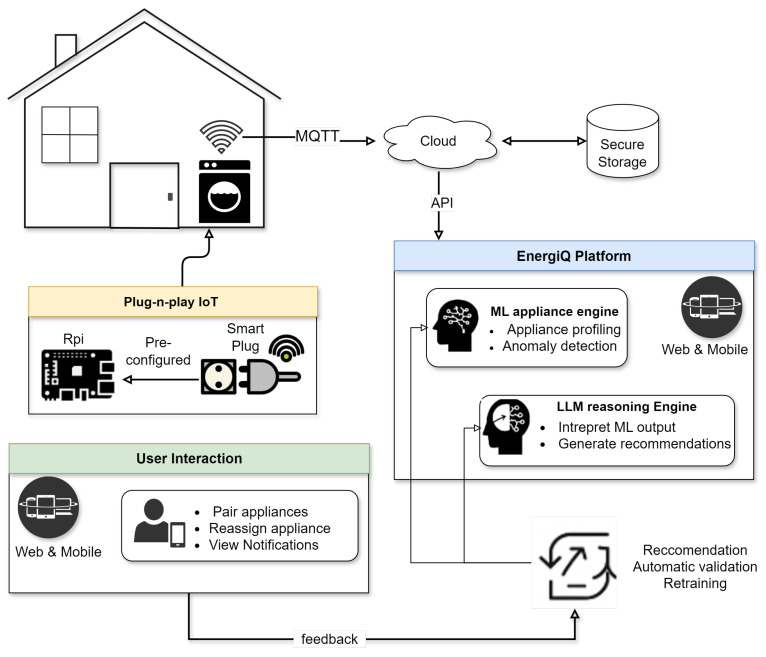
EnergiQ overview: a unified pipeline from IoT-based data acquisition to LLM-driven reasoning and user interaction.

As the system observes home appliance operation and usage over time, its machine learning engine builds detailed consumption profiles and detects anomalies, such as inefficiencies or behavioral shifts. When such events occur, EnergiQ’s LLM reasoning engine translates technical findings into natural-language explanations and context-aware recommendations, helping users make informed decisions, like adjusting usage or checking for faults. EnergiQ includes a feedback loop, where users can confirm or correct system interpretations (e.g., when a device is replaced), enabling automatic retraining and continual model refinement. This makes the system adaptive to real-world variability in appliance behavior. An overview of the EnergiQ platform’s components and data flow is illustrated in Figure 1, which shows the interaction between IoT sensing, cloud processing, ML/LLM analytics, and user engagement interfaces.

**Figure 2:**
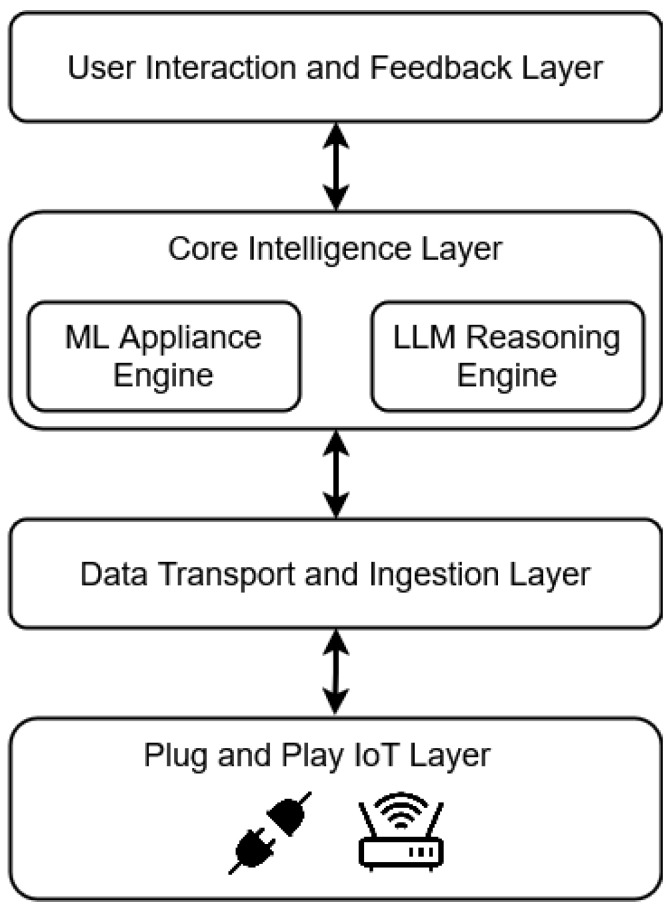
EnergiQ system top-down architecture showing data flow from device sensing to user feedback.

The architecture of EnergiQ is layered and hierarchical to facilitate effective real-time data exchange, user-friendly interaction, and smart analytics. As shown in Figure 2, the architecture of the four fundamental layers consists of the Plug-and-Play IoT Layer, the Data Transport and Ingestion Layer, the Core Intelligence Layer, and the User Interaction and Feedback Layer. Such an architecture facilitates high configurability in home settings, as well as seamless data flow and modularity in scalability.

And at its core is the **Plug-and-Play IoT Layer**, collecting appliance-level energy information. Standalone sensing agents are devices such as Raspberry Pi-based sensors and smart plugs, which capture high-resolution electrical signals, voltage, current, frequency, and power usage. The devices need not be set up and start sending data the moment they are deployed, with simple installation and extreme scalability.

On top of this, the **Data Transport and Ingestion Layer** provides secure and reliable data delivery into the cloud. With MQTT, a low-latency IoT-tuned protocol, communication remains low-latency even in bandwidth-constrained places. The data is structured upon receipt, stored in time-series databases, and fetched by RESTful APIs to analytics and interface modules for processing.

At the center is the **Core Intelligence Layer** consisting of two highly integrated engines. The Machine Learning Appliance Engine analyzes time-series data to create consumption profiles, identify behavioral abnormalities, and categorize appliance types based on XGBoost. Supportive to it is the LLM Reasoning Engine interpreting these analytical results into natural language and creating personalized explanations, alerts, and actionable recommendations. These engines are tightly integrated with internal APIs for smooth coordination from statistical inference to user-level communication.

On top of that is the **User Interaction and Feedback Layer**, granting users seamless interaction through web and mobile channels. Users can tag devices with smart plugs, view real-time usage, and gain system-provided insights. Most importantly, perhaps, this layer grants an interactive feedback loop: when users affirm or modify system perception, their feedback is fed back to the intelligence layer, enabling ongoing model refinement and learning.

Across the system, communication systems are systematic and two-way. Data is carried upwards from sensors to the layer of intelligence for analysis, and information is passed downwards again to users in natural language. User input, at the same time, passes downwards to drive model accuracy. The ongoing, closed-loop process not only drives system performance as well as personalization but also promotes transparency as well as trust by reducing complex analytics down to simple, human-understandable guidance.

### 3.2. Proposed EnergiQ Platform

The EnergiQ system is constructed around a service-oriented, modular architecture that supports scalability, maintainability, and high-performance real-time capability within the smart home energy ecosystem. The system comprises a Laravel backend, a Vue.js front end, PostgreSQL for holding structured data, and auxiliary Python-based microservices to support machine learning inference as well as IoT data consumption. All the components communicate with each other via secure and documented APIs, protected using HTTPS with TLS encryption and token-based authentication (e.g., JSON Web Tokens), ensuring authorized and encrypted access to the backend services.

In the backend, EnergiQ employs the Laravel PHP framework to manage application business logic, API routing, and auth functions. Laravel’s queue system using Redis manages asynchronous workloads such as telemetry processing, ML service requests, anomaly analysis, and alert generation. API endpoints are made available through a RESTful interface that is secured by Laravel Sanctum, enabling secure token-based interaction between client apps and server systems. Facing the front-end, the system uses Vue.js to present a responsive Single Page Application (SPA) that enables users to allocate appliances to smart plugs, live track usage, receive smart alerts, and give feedback. Axios manages asynchronous HTTP communication, and Vuex manages centralized state management of predictable UI behavior between application views.

The platform’s structured data is managed using PostgreSQL, which serves as the primary relational database for storing user information, appliance-device mappings, consumption logs, anomaly events, and feedback entries. The database schema is normalized and indexed to support efficient querying of time-series and relational data. Laravel’s Eloquent ORM allows expressive database interaction with transactional consistency and code organization that is simple to maintain. Historical reporting and real-time decision-making are facilitated by the central data layer, which is the foundation of EnergiQ’s analytics pipeline.

For device level telemetry ingestion, EnergiQ uses MQTT-based architecture optimized for low latency and lightweight communications. Smart plugs and Raspberry Pi gateways publish the data, power, voltage, and frequency to an MQTT broker.A Python subscriber service listens on such topics and performs validation by checking the structure of incoming payloads (e.g., via JSON schema validation), verifying timestamps for freshness, and authenticating device IDs to ensure data integrity and prevent malformed or spoofed messages. This pattern decouples the ingestion pipeline from the rest of the application by using a Redis-backed message queue. Incoming MQTT messages are published to the queue and processed asynchronously by consumer services. This ensures that data ingestion remains non-blocking and responsive, even when handling high-frequency telemetry streams from multiple devices in parallel.

The platform’s machine learning component is also developed in Python 3.13.0 and runs as an external microservice. It presents RESTful endpoints to the backend to conduct device classification and anomaly detection with models like XGBoost and CNN-LSTM autoencoders. When new telemetry becomes available, Laravel asynchronously sends it to the ML service, which returns structured diagnostic outputs and stores them, and optionally pushes them to the user interface for alerting or explanation.

To connect technical model outputs to human-comprehensible intelligence, EnergiQ includes a LLM reasoning service within its system. This module processes the output of the ML engine and translates it to natural language recommendations, reminders, and energy-saving tips that are behavior-specific for residences. The LLM is augmented by Retrieval Augmented Generation (RAG) by retrieving a response from a FAISS-based vector store that holds appliance metadata, previously observed usage patterns, and domain context data. This allows the system to produce output that not only makes sense but is also personalized, timely, and pertinent to the specific energy profile of each user.

#### Data Handling and Privacy Issues

EnergiQ is designed on a privacy-by-design approach, leveraging edge-based computation to protect user data from the very beginning. Data is pre-processed first by local gateways (Raspberry Pi), anomalies are detected, and aggregation happens before only necessary anonymized results are streamed to the cloud. Encrypted MQTT protocols transfer data, and access is strictly managed with token-based authentication (Laravel Sanctum) [27]. No personally identifiable information is collected or retained; rather, the system depends on pseudonymization, data minimization, and limits data storage to what is absolutely needed for energy profiling, anomaly detection, and feedback generation. Only the necessary, aggregated, and anonymized summaries are sent to the cloud, ensuring no raw or personally identifiable information (PII) leaves the user’s premises [28].

The LLM reasoning layer operates solely on metadata and aggregated behavioral profiles through Retrieval-Augmented Generation (RAG), without access to raw consumption data or user identities. This ensures that personalized recommendations remain privacy-compliant [29]. The platform maintains transparency through clear user controls and offers continuous learning mechanisms without compromising data protection.

### 3.3. Machine Learning for Consumption Pattern Validation

In this section, the machine learning methodology used by the EnergiQ platform to validate and interpret appliance-level energy consumption patterns is described. More specifically, the use of statistical feature-based classification for device recognition, a hybrid deep learning approach for anomaly detection, and a user-driven retraining mechanism to ensure continual adaptation are illustrated.

#### 3.3.1. Device Detection Using Statistical Features

To accurately detect and classify appliances, the EnergiQ platform employs a machine learning framework based on statistical features extracted from time-series power consumption data. These features form a numerical representation of the appliance’s behavioral signature and are critical for distinguishing different types of devices using only plug-level energy data. The following features are extracted from the power signal and used as input for classification.

Mean Power: The mean represents the average consumption of an appliance over a given time window:

(1) $$\mu =\frac{1}{N}\sum_{i=1}^{N}P_{i}$$

where $P_{i}$ is the power at time index *i*, and *N* is the total number of samples in the time window. This feature is useful for broadly separating high-power devices such as ovens or water heaters from low-power or idle appliances like routers and LED lights. It provides an overall energy baseline.

Standard Deviation: The standard deviation captures the variability in power usage:

(2) $$\sigma =\sqrt{\frac{1}{N}\sum_{i=1}^{N}{\left(P_{i}-\mu \right)}^{2}}$$

where μ is the mean power. A low standard deviation indicates stable operation (e.g., a constantly on device), while a high standard deviation points to devices with multiple internal states or cycling behavior, such as washing machines or air conditioners.

Peak-to-Average Ratio (PAR): PAR emphasizes the presence of sharp peaks in power consumption: 

(3) $$\mathrm{PAR}=\frac{\max(P)}{\mu }$$

where max(P) is the highest recorded power in the window. This is particularly useful for detecting appliances like kettles, toasters, or hair dryers, which consume large amounts of power in short bursts, resulting in a high PAR.

Rolling Mean: The rolling mean is a localized version of the average:

(4) $${\mathrm{RM}}_{w}\left(t\right)=\frac{1}{w}\sum_{i=t-w+1}^{t}P_{i}$$

where *w* is the size of the sliding window, and *t* is the current time index. It helps identify slow variations in usage patterns, such as the gradual warming of an oven or temperature regulation in a heating system. Different time scales (e.g., 30, 60, 90 min) help capture both short- and long-term behavior.

Rolling Standard Deviation: Measures localized fluctuations:

(5) $${\mathrm{RSD}}_{w}\left(t\right)=\sqrt{\frac{1}{w}\sum_{i=t-w+1}^{t}{\left(P_{i}-{\mathrm{RM}}_{w}\left(t\right)\right)}^{2}}$$

where ${\mathrm{RM}}_{w}\left(t\right)$ is the rolling mean at time *t*. This is useful for detecting devices with variable consumption over short intervals, such as washing machines going through different cycles (wash, rinse, spin) or HVAC systems toggling on and off frequently.

Entropy: Entropy quantifies the level of disorder or unpredictability in the signal:

(6) $$H=-\sum_{j=1}^{B}p_{j}\log\left(p_{j}\right)$$

where $p_{j}$ is the probability that the signal falls into the *j*-th bin, and *B* is the total number of bins. Appliances with regular, predictable usage (e.g., a refrigerator cycling every 30 min) will have lower entropy, while irregular behavior (e.g., manual use of a microwave) increases entropy. This helps differentiate between autonomous and user-driven appliances.

Skewness: Skewness captures the asymmetry of the distribution:

(7) $$\mathrm{Sk}=\frac{\frac{1}{N}\sum_{i=1}^{N}{\left(P_{i}-\mu \right)}^{3}}{\sigma^{3}}$$

where μ is the mean and σ is the standard deviation. Positive skew suggests rare, large power draws (common in bursty devices like kettles), whereas negative skew may indicate sustained higher usage. It helps in classifying how energy is distributed over time.

Kurtosis: Measures the “tailedness” or concentration of power readings:

(8) $$K=\frac{\frac{1}{N}\sum_{i=1}^{N}{\left(P_{i}-\mu \right)}^{4}}{\sigma^{4}}$$

A high kurtosis value means most of the time the appliance is off or idle, with short, high-activity bursts. Low kurtosis reflects more balanced activity. This distinction is especially useful for separating devices that idle often from those with continuous operation.

Proportion Above Threshold: Quantifies how often the device is active:

(9) $$P_{\mathrm{above}}=\frac{1}{N}\sum_{i=1}^{N}⊮\left(P_{i}>T\right)$$

where $⊮(P_{i}>T)$ is 1 if the power at time *i* exceeds a defined threshold *T*, otherwise 0. This feature helps identify always-on or standby devices (e.g., freezers) versus those with sporadic use (e.g., hair dryers), based on their on-time duration.

Periodogram Peaks: Used to capture frequency-based patterns in the signal:

(10) $$\mathrm{PSD}\left[k\right]=\frac{1}{N}{\left|\sum_{n=0}^{N-1}P_{n}e^{-j2\pi nk/N}\right|}^{2}$$

where PSD[k] represents the power spectral density at frequency index *k*. This feature is important for detecting periodic appliances like fridges or HVAC systems, which operate in consistent on-off cycles. The top five dominant frequencies are retained as feature components.

Matrix Profile: Identifies recurring motifs or subsequences in the signal:

(11) $${\mathrm{MP}}_{m}\left[i\right]=\min_{j\neq i}\sqrt{\sum_{k=0}^{m-1}{\left(P_{i+k}-P_{j+k}\right)}^{2}}$$

where *m* is the length of the subsequence, and $MP_{m}\left[i\right]$ is the distance to its nearest neighbor. This feature is powerful for detecting devices with repeating operational behavior (e.g., washing machines with consistent program cycles). It captures self-similarity that standard statistics might miss.

A summary of the extracted statistical features, along with the purpose for which each feature was selected, is presented in Table 3.

To utilize the extracted features for device recognition, an XGBoost classifier is used. Given an input feature vector $x\in R^{d}$ derived from the statistical features described above, XGBoost applies an ensemble of *K* decision trees to predict the class label $\hat{y}$ of the device:

(12) $$\hat{y}=\sum_{k=1}^{K}f_{k}\left(x\right),\;f_{k}\in F$$

where $f_{k}$ is the *k*-th regression tree in the ensemble and F is the space of all possible trees.

XGBoost builds the model in an additive manner, optimizing an objective function that includes both a loss term and a regularization term:

(13) $$\;L=\sum_{i=1}^{n}l\left(y_{i},{\hat{y}}_{i}\right)+\sum_{k=1}^{K}\Omega \left(f_{k}\right)$$

where $l(y_{i},{\hat{y}}_{i})$ is a differentiable loss function (e.g., softmax for classification), and $\Omega (f_{k})$ is a regularization term that penalizes model complexity to prevent overfitting.

Each tree $f_{k}$ is trained to minimize the residual errors from the previous stage using gradient boosting. This allows the model to learn non-linear relationships between features such as power variability, periodicity, or entropy, and the corresponding device class.

The XGBoost classifier is particularly well suited to this task due to its robustness, efficiency, and ability to handle heterogeneous feature types. It performs well even with relatively small amounts of labeled data and generalizes effectively across diverse appliance categories.

#### 3.3.2. Anomaly Detection Through Hybrid Autoencoder-Based Monitoring

**Figure 3:**
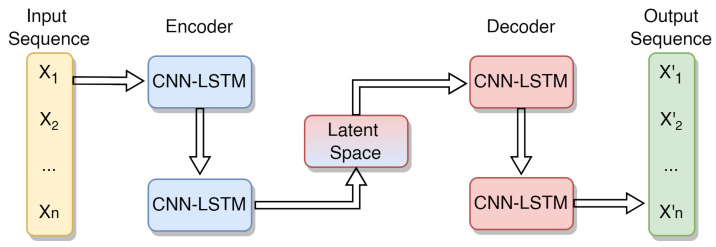
CNN-LSTM autoencoder architecture.

To complement the classification of appliance types and ensure the detection of abnormal consumption patterns, the EnergiQ platform incorporates an anomaly detection mechanism. This mechanism is designed to identify deviations from normal operational behavior that may indicate appliance faults, inefficiencies, or misuses. Figure 3 illustrates the proposed autoencoder.

##### Encoding Process

Given a multivariate time-series input $X\in R^{T\times d}$, where *T* is the number of time steps and *d* is the number of input features, the encoder applies 2D convolutional filters to capture both temporal and inter-feature patterns. Each feature sequence is treated as a spatial column and time as the row dimension.

The 2D convolution is computed as follows:

(14) $$C_{t,f}=\phi \left(\sum_{i=0}^{k_{t}-1}\sum_{j=0}^{k_{f}-1}W_{i,j}\cdot X_{t-i,f-j}+b\right)$$

where *W* is the convolution kernel of size $k_{t}\times k_{f}$, *b* is a bias term, and ϕ(·) is a nonlinear activation function such as ReLU.

The CNN layers reduce the spatial-temporal dimensionality and extract local patterns, which are then passed into stacked LSTM layers to model temporal dependencies. The LSTM cell is updated as follows:

(15) $$\begin{array}{cc} f_{t} & =\sigma (W_{f}x_{t}+U_{f}h_{t-1}+b_{f}) \end{array}$$

(16) $$\begin{array}{cc} i_{t} & =\sigma (W_{i}x_{t}+U_{i}h_{t-1}+b_{i}) \end{array}$$

(17) $$\begin{array}{cc} {\tilde{c}}_{t} & =\tanh(W_{c}x_{t}+U_{c}h_{t-1}+b_{c}) \end{array}$$

(18) $$\begin{array}{cc} c_{t} & =f_{t}⊙c_{t-1}+i_{t}⊙{\tilde{c}}_{t} \end{array}$$

(19) $$\begin{array}{cc} o_{t} & =\sigma (W_{o}x_{t}+U_{o}h_{t-1}+b_{o}) \end{array}$$

(20) $$\begin{array}{cc} h_{t} & =o_{t}⊙\tanh\left(c_{t}\right) \end{array}$$

where, $x_{t}$ is the input to the LSTM at time *t*, $h_{t}$ is the hidden state, $c_{t}$ is the memory cell, and σ is the sigmoid activation. Additionally, the symbol ⊙ is used to indicate element-wise multiplication.

##### Decoding and Reconstruction

The decoder reverses the process using LSTM layers followed by 2D transpose convolution layers to reconstruct the original input shape. The decoder LSTM generates hidden states ${\hat{h}}_{t}$, and the output is reconstructed as follows:

(21) $${\hat{X}}_{t,f}=\psi \left(\sum_{i=0}^{k_{t}-1}\sum_{j=0}^{k_{f}-1}{\hat{W}}_{i,j}\cdot {\hat{h}}_{t-i,f-j}+\hat{b}\right)$$

where $\hat{W}$ and $\hat{b}$ are the transpose convolution weights and biases, and ψ is the ReLU activation function.

The model is trained to minimize the reconstruction error using the Mean Squared Error (MSE) loss: 

(22) $$L_{\mathrm{MSE}}=\frac{1}{T\cdot d}\sum_{t=1}^{T}\sum_{f=1}^{d}{\left(X_{t,f}-{\hat{X}}_{t,f}\right)}^{2}$$

##### Anomaly Scoring and Detection

At inference time, for each new input *X*, the model produces a reconstruction $\hat{X}$, and the Mean Absolute Error (MAE) is computed as follows:

(23) $$\mathrm{MAE}\left(X,\hat{X}\right)=\frac{1}{T\cdot d}\sum_{t=1}^{T}\sum_{f=1}^{d}\left|X_{t,f}-{\hat{X}}_{t,f}\right|$$

A threshold is used to determine if the error indicates an anomaly:

(24) $$\mathrm{Threshold}=\mu_{\mathrm{MAE}}+\alpha \cdot \sigma_{\mathrm{MAE}}$$

where $\mu_{\mathrm{MAE}}$ and $\sigma_{\mathrm{MAE}}$ are the mean and standard deviation of the reconstruction error on normal training data, and α is a sensitivity factor.

Through preliminary tuning, a value α = 3 was found as optimal and provided a good trade-off between precision and recall across appliance types. Lower values (e.g., α = 2.5) increased sensitivity but also led to a higher false positive rate, reducing the F1-score. In contrast, α > 3 overly suppressed anomaly detection.

This CNN-LSTM autoencoder architecture is well suited for detecting complex anomalies in multivariate features related to energy consumption patterns. The 2D CNN layers effectively capture inter-feature correlations, while the LSTM layers model temporal dynamics. The model is capable of detecting irregular operating cycles, unexpected spikes, or drops in consumption, as well as long-term drift or degradation in appliance behavior.

While the CNN-LSTM autoencoder achieved promising results, its performance can vary depending on appliance heterogeneity. Devices with highly irregular or user-driven behavior created challenges due to less predictable patterns, leading to higher reconstruction error variance. To mitigate this, the model was trained separately on clustered groups of appliances with similar temporal profiles, and adjusted the anomaly thresholds dynamically using appliance-specific baseline statistics. Additionally, dropout regularization and early stopping were applied during training to reduce overfitting on high-frequency devices. These steps improved generalization across diverse appliance types while maintaining sensitivity to meaningful anomalies.

#### 3.3.3. Model Retraining and Continuous Learning

To ensure long-term adaptability, the EnergiQ platform employs a dynamic retraining mechanism driven by user feedback. The initial deployment leverages pre-trained models developed using historical data from appliance datasets with similar profiles. However, EnergiQ is designed to operate proactively: as soon as the system is connected, it begins collecting real-time data and validating user feedback to continuously adapt to the specific characteristics of each household’s appliances. This ensures that model performance improves over time through in situ learning.

Whenever a user confirms a significant change in appliance behavior, such as a replacement or reconfiguration, the system marks the corresponding data segment as validated. These labeled instances are then appended to the training dataset for future learning cycles.

Once a sufficient volume of confirmed feedback is collected, the machine learning models are retrained to incorporate the updated behavior patterns. The XGBoost-based device classifier is updated using newly validated statistical features to reflect evolving usage features. Simultaneously, the CNN-LSTM autoencoder is fine-tuned using new sequences of normal appliance operation, ensuring that its learned reconstruction baselines remain accurate.

To prevent overfitting and preserve historical knowledge, transfer learning techniques are applied: previous model weights are retained and incrementally adjusted rather than replaced entirely. Prior to deployment, updated models are validated on a hold-out dataset and promoted only if they meet or exceed predefined thresholds in accuracy and F1-score.

Retraining is initiated either periodically (weekly) or when a sufficient number of validated user feedback events, 10 samples, is collected. This hybrid trigger ensures timely adaptation without overfitting to sparse or anomalous updates.

By linking model updates directly to user-confirmed behavior, EnergiQ maintains a responsive and intelligent feedback loop. This allows the platform to remain accurate over time, continuously improving its ability to classify appliances and detect anomalies in real-world residential settings.

### 3.4. Role of Large Language Models

LLMs on the EnergiQ platform establish the essential missing link between sophisticated ML analytics and natural, conversational interaction. Traditional ML models used within EnergiQ are highly proficient in analyzing time-series energy data, recognizing appliance-specific behavior, and recognizing anomalous use behavior. They have the propensity to report their findings in meaningless numbers, clusters, or statistical anomalies representations that are irrelevant to and automatically dismissed by non-expert users.

LLMs fill this gap by serving not just as natural language generation interfaces but also as wise counselors. LLMs simplify unclear ML outputs into lucid, human-readable insights, providing natural language explanations, context-dependent warnings, and personalized, actionable energy-saving advice. This ability is at the heart of the EnergiQ mission: equipping consumers with timely, simple-to-grasp, and behaviorally appropriate feedback. The LLM collaborates with the overall system architecture in enabling an interactive feedback loop to read data, suggest action, and continuously adapt to user input.

Aside from interpretation, the LLM layer is also trained with domain-specific guidance and RAG, enabling it to access dynamic contextual data such as time-of-use rates, appliance labels, and historical usage patterns and adapt its output to address the individual needs of each home.

#### 3.4.1. Dataset Creation and Structure

To adapt the LLM for interpreting appliance-level energy data, a dataset was created based on domain-expert recommendations [30]. The dataset is based on established best practices for energy efficiency and is publicly accessible via Zenodo [31]. It includes 100 real-world energy usage scenarios, selected to represent a diverse range of practical contexts, providing the necessary data for fine-tuning the model. Each entry contains the following:

1. Recommendation Each instance includes a device-related error context (e.g., ’fridge door open’) and a corresponding recommendation for corrective action.
2. Device and Error (Input_Real): This feature includes data related to the device type, derived from the smart plug, as well as detected anomalies identified by the machine learning components described in Section 3.3.
3. Frequency: This feature is a frequency label that indicates the commonality of the issue, categorized as High, Medium, or Low.

These templates enabled automatic creation of linguistically diverse and context-sensitive advice. The final dataset was serialized in JSONL format to support integration with instruction tuning pipelines, as summarized in Table 4.

This structure allows the model to learn mappings between the type of anomaly detected (e.g., “Door open”, “More spikes”, “Different duration”) and the appropriate behavioral advice, contextualized by device type and error severity. The diversity and granularity of examples ranging from mechanical faults to user-driven inefficiencies equip the model with the ability to produce rich, actionable, and personalized responses grounded in energy domain knowledge. The dataset is also representative of typical appliance behaviors such as compressor overload, thermal inefficiency, or out-of-normal use (e.g., constant opening and closing of doors), so that the LLM can learn realistic household energy patterns.

Shown in Table 5 is the recommendation dataset, summarizing each column’s purpose and indicating the number of non-null entries present for fields such as recommendations, inferred consumption patterns, real-world input triggers, and frequency labels.

#### 3.4.2. Instruction-Tuning Methodology

In order to allow the LLM on the EnergiQ platform to generate correct, context-based energy saving advice, both instruction tuning and RAG were experimented with. The model was first instruction-tuned with a domain-specific set of prompt response frequency triplets to align it with appliance-level diagnosis and energy advisory lexicon. At inference time, RAG allows the model to fetch fresh contextual data like appliance information, past usage habits, and time of use electricity prices from an external knowledge store. This guarantees that recommendations not only be well formed but also dynamically adapted to each household’s unique conditions and energy behavior.

Each prompt was derived from the machine learning outputs and includes the following:

- Device type (e.g., fridge, heater)
- Anomaly classification (e.g., door open, more spikes, different duration)
- Usage context (optional: time of day, recurring pattern)

Example prompt format:

Device: fridge
Error: Door open

Responses were generated based on a structured dataset of appliance-level energy recommendations, which systematically maps specific device anomalies to corresponding energy-saving actions. Each response targeted one or more of the following:

- Energy Efficiency: Advice on reducing waste (e.g., minimize door-open time, avoid overcooling).
- User Behavior Optimization: Suggestions addressing frequent or irregular use.
- Preventive Maintenance: Tips to preserve device performance and avoid faults.

Example response:

*Avoid leaving the fridge door open for extended periods. This can increase energy use and force the compressor to work harder, reducing its lifespan.*

Building on this foundation, the instruction-tuning task was cast as conditional generation, where the LLM is trained to produce natural language responses conditioned on structured prompts. The loss was computed using cross-entropy over token sequences, comparing the generated output to the target recommendation.

To improve generalization and reduce redundancy in outputs, the training set included multiple variations in each recommendation. Techniques such as synonym substitution, paraphrasing, and sentence restructuring were employed. This variation exposed the model to a richer set of expressions, enhancing fluency, diversity, and end-user relatability.

This methodology ensures the LLM not only understands the semantic mapping between energy behavior and advice but also maintains a clear, helpful, and non-technical communication style appropriate for residential users.

#### 3.4.3. Retrieval Augmented Generation (RAG)

RAG is a hybrid framework that enhances generative language models by incorporating external knowledge retrieved dynamically from a large corpus or database. This approach improves factual accuracy and contextual relevance by grounding generation on retrieved data relevant to the input query.

The process involves three main components:

1. **Query Embedding:** Given a user query *q*, a pre-trained embedding model maps it into a dense vector space:
  $$e_{q}=\mathrm{Embed}\left(q\right),\;e_{q}\in R^{d},$$
  where *d* is the embedding dimension. In the implemented system, this is realized using a Sentence-BERT model.
2. **Retrieval:** The query embedding $e_{q}$ is compared against a database of embeddings ${\left\{e_{i}\right\}}_{i=1}^{N}$, representing the knowledge base entries. The system retrieves the index $\hat{i}$ of the most similar entry by maximizing a similarity function, such as the negative Euclidean distance:
  $$\hat{i}=\arg\min_{i}{∥e_{q}-e_{i}∥}_{2},$$
  or equivalently maximizing cosine similarity:
  $$\hat{i}=\arg\max_{i}\frac{e_{q}\cdot e_{i}}{∥e_{q}∥∥e_{i}∥}.$$
  The FAISS library is used for an efficient nearest neighbor search in high-dimensional spaces.
3. **Contextual Generation:** The retrieved document or entry $d_{\hat{i}}$ provides contextual information for the generative model. The generation model produces a response *r* conditioned on the original query and the retrieved context:
  $$r=\mathrm{Generate}(q,d_{\hat{i}}).$$
  This is modeled probabilistically as follows:
  $$P\left(r|q,d_{\hat{i}}\right)=\prod_{t=1}^{T}P\left(r_{t}|r_{<t},q,d_{\hat{i}}\right),$$
  where $r_{t}$ is the generated token at step *t*.

In practice, user queries are first standardized using a formatting function that combines anomaly descriptions and priority levels. Sentence embeddings for all dataset entries are precomputed and indexed using FAISS to support fast nearest-neighbor retrieval. When a query is received, the system identifies the most relevant entry from the database and incorporates it into a prompt sent to a language model (e.g., via the Ollama API). The result is a context-aware, personalized energy-saving recommendation. This architecture enables efficient and effective generation of context-aware recommendations by leveraging both retrieval and generative capabilities.

## 4. System Implementation and Use Cases

### 4.1. Platform Deployment

The implementation of the EnergiQ platform aims to give the user an intuitive and smart experience of discovering and managing their appliance-level energy use. The second a user puts a smart plug into an appliance, the platform starts a number of automated, smart processes that transform raw data into actionable, personalized energy insight.

Once the users get the system, they start by inserting a smart plug between an appliance at home (like a washing machine) and a socket. The smart plug captures detailed electrical attributes, including voltage, current, frequency, and active power, at a granularity of one-minute intervals. This sampling frequency strikes a balance between capturing the characteristic behavior of household appliances and minimizing unnecessary data volume, aligning with established best practices in residential energy monitoring [30]. This is recorded with time-stamping and streaming in real time to a nearby data agent, a Raspberry Pi, which also serves as a gateway to the home setting and the EnergiQ environment. In the background, the local agent communicates using the MQTT protocol to pass on this data in real time to the core system, which resides in the cloud. The MQTT stack provides the assurance that even in the case of low bandwidth or unstable connectivity, the data is stable and intact. In the cloud, the platform’s REST APIs publish this data to the internal services that are tasked with the core analysis, as well as the web and mobile apps used by the user.

The more the user utilizes their appliances, the more the platform starts to refine its interpretation of individual usage patterns. The machine learning algorithm, which incorporates time-series models such as DenseNet-1D and LightGBM, is pre-trained on general consumption patterns using publicly available datasets including REFIT and UK-DALE, covering a range of appliance types [32,33]. This enables the system to easily accommodate the unique situation of the user’s household. Within a matter of mere cycles of use, it can build a pattern of behavior for each device and accurately report what “normal” use appears as for each appliance.

When the platform identifies something unusual, say, when the user installs a new, more efficient washing machine in place of the old one, the system identifies a change in behavior. The LLM engine steps in here. Rather than presenting a straightforward technical alert, it creates a natural-language alert such as follows:

*“We’ve noticed that your washing machine’s consumption pattern has changed significantly. Did you replace the device?”*

The user is answered back with this message through the EnergiQ web or mobile app, where the user can accept or reject the system’s interpretation. This feeds back to the active learning loop of the platform to improve the accuracy of future analysis directly. As the system aggregates more data over time, it makes more insightful observations. It may inform the user, say, that the refrigerator has begun exhibiting initial signs of malfunctioning due to excessive use. Or it would recommend running the dishwasher at odd hours in order to save energy. These are translated automatically to match the user’s patterns of use, the hour of the day, and potentially time-of-use electricity prices all made understandable by the LLM reasoning layer.

**Figure 4:**
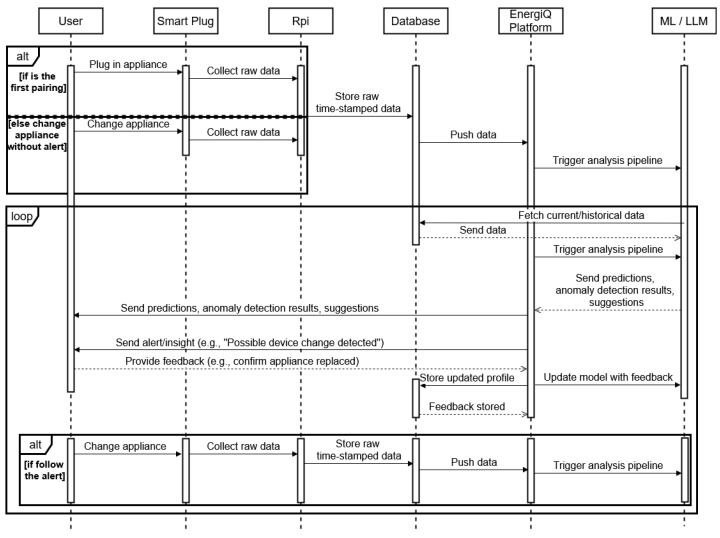
Sequence diagram of EnergiQ system.

The sequence diagram shown in Figure 4 graphically illustrates the whole operational life cycle of the EnergiQ platform varies from device mounting to smart user feedback. It starts with the user on-off or replacing the device, which makes the smart plug start capturing detailed electrical information. The raw data is time-stamped and sent to the local Raspberry Pi, which sends it to the cloud database via the MQTT protocol. The EnergiQ system captures this information and calls its analysis pipeline, combining machine learning and LLM reasoning to search for patterns of use and anomalies. If anomalies are found, e.g., a likely device replacement, the system creates natural language notifications and presents them to the user through the interface. The user response acceptance or rejection of the insight is fed back into the system to update profiles and retrain models so that the system learns and adapts continuously. This feedback cycle of sensing, analysis, alerting, and adaptation, as shown in the diagram, represents the intelligent, user-driven architecture of the EnergiQ platform.

### 4.2. Platform Functionality and Design Principles

The EnergiQ system is designed with two goals of technical adequacy and user-friendly simplicity to enable straightforward interaction with both the user and home energy data. Central to the system is appliance-level real-time monitoring, contextual pattern inspection, and aware guidance, all presented to the user through an extremely responsive interface.

EnergiQ broadens its fundamental capabilities to enable appliance-level profiling, anomaly reporting, and adaptive system response. Through the integration of machine learning and big language models, it tracks and reports running operational information like irregular usage patterns or device malfunctions. The system offers a back-channel interface that responds when users confirm or correct system interpretation and redirects appliances such that tracking and analysis accuracy continuously improve in different home environments. The platform also features an active learning loop, allowing users to accept or reject system conclusions, rename appliances, and receive personalized energy-saving recommendations. Access to the system is available across both web and mobile platforms, ensuring flexibility and convenience for users managing multiple devices.

The design of EnergiQ follows several core principles. Its plug-and-play smart plugs are simple to install, requiring no technical background, which enhances user adoption and scalability. The system’s architecture is modular and scalable, with independently upgradable components for data acquisition, processing, reasoning, and user interaction. Resilience under low-bandwidth conditions is ensured through the use of MQTT communication protocols. EnergiQ is also developed with privacy and security in mind, securely handling data and abstracting user or appliance details where necessary. Finally, the integration of natural language explanations via LLMs fosters transparency and builds user trust by making machine learning outputs easily understandable.

### 4.3. Use Case Scenarios

This section describes important scenarios illustrating how EnergiQ tracks appliance usage, identifies anomalies, and provides insights in natural language. Each use case illustrates the platform’s capability to provide precise energy monitoring and actionable feedback with minimum user effort.

#### 4.3.1. Use Case 1: Normal Appliance Operation

This is the control scenario where a domestic appliance is operating as expected, always on through a smart plug, and giving good telemetry data to the EnergiQ system. The appliance is acting in terms of usage that is consistent with past behavior and shows no indication of faults or drift in behavior. The machine learning algorithms show no anomalies, and the LLM classifies this as normal operation. In the UI, the device is displayed as green status indicator, and a passive state event like “Device operating within expected parameters” is recorded. No alarm is triggered, and no user action is solicited, so an unobtrusive user experience is enabled. EnergiQ stores this quietly in the background for future learning and retrospective analysis in order to provide absolute continuity of the device behavior history.

#### 4.3.2. Use Case 2: Device Replaced Without User Notification

This use case is the scenario when a user replaces a device plugged into a smart plug without a new configuration of the EnergiQ platform. The system, as an ongoing energy consumption profile watchdog, realizes that there is a drastic deviation from the established profiles beforehand. The machine learning module recognizes the deviation as a probable device replacement. The LLM reasoning layer builds a natural language query like: “We have noticed a significant change in energy usage. Did you recently replace the appliance connected to this smart plug?” This notification is presented on the user interface, prompting the user to approve or edit the appliance identity. On approval, the system resets the device profile and starts learning the new consumption pattern. Such an occurrence proves EnergiQ can identify hardware changes automatically without direct user input, allowing appliance-level monitoring accuracy and reliability to be maintained in a consistent manner.

#### 4.3.3. Use Case 3: Abnormal Consumption Suggests Fault

Here, a device still functions but starts behaving differently from its typical consumption pattern, like long cycles, random power surges, or unexpected activity. The machine learning platform detects the anomaly and marks the activity as suspicious. The LLM produces an easy-to-understand alert; for instance, “Your fridge is using more power than normal, and this could be a sign that something is amiss. Would you like to investigate?” The user is informed about this from the platform interface. If it is accepted, a log is created and additional monitoring can be initiated. This illustration is how EnergiQ is applied in proactive maintenance, where faults are signaled early before breakdown, increasing appliance life and energy efficiency.

#### 4.3.4. Use Case 4: Unauthorized or High-Load Appliance Detected

This scenario arises when a new, power-seeking device is connected to a smart plug without prior registration or identification. The platform detects an unusual, rising energy discrepancy that does not correspond to any known appliance model. The LLM responds with something like, “A power-seeking device has been connected to this port. What kind of appliance is it?” This alert anticipates unauthorized or potentially hazardous equipment from getting out of sight. The user can opt for naming the appliance, disabling the warning, or powering off the device. Here, it is worth observing that it highlights EnergiQ’s capability to stay vigilant and in control of the home energy situation to prevent the shock of escalated energy bills or circuit overload.

#### 4.3.5. Use Case 5: Energy Optimization Suggestion Based on Usage Patterns

This use case is realized when EnergiQ detects that an appliance is being used fairly, often at peak tariff times. In the absence of fault or anomaly, the system detects an opportunity for energy and cost savings. The LLM reasoner engine formulates a context-specific recommendation like, “We have seen that your dishwasher operates most frequently between 6 and 9 p.m. Operating it after 10 p.m. may lower your electricity bill.” The proposal is made as a non-intrusive proposal within the user interface. This shows that EnergiQ is not only capable of identifying problems, but also of providing proactive value-added propositions in favor of smart energy consumption and user behavior.

#### 4.3.6. Use Case 6: Appliance Left on Unexpectedly

EnergiQ picks up on the fact that an appliance like an oven or water heater has been running for a whole lot longer than it possibly should have. The ML model identifies it as a probable neglect or safety problem. The LLM crafts a prompt such as follows: “Your water heater has been operating for 5 h without a break, longer than usual. Shall you turn it off?” The warning is called out to the user via the mobile application, perhaps with an action button for prompt response. This usage case illustrates how EnergiQ increases awareness and safety by intelligent pattern observation.

## 5. Experimental Results

### 5.1. Experimental Set Up

To evaluate the effectiveness and usability of the EnergiQ platform in real-world conditions, a longitudinal field deployment across 20 residential apartments over a period of 6 to 8 months was conducted. Each apartment was equipped with the following hardware configuration: 1 Raspberry Pi 4 model B [34] acting as a local edge gateway and four pre-configured smart plugs (i.e., Fibaro smart plugs [35]). The system is designed for plug-and-play deployment, requiring no technical setup. All devices were pre-flashed and networked, allowing users to start immediately.

Before starting, participants completed a brief pre-study questionnaire to assess their baseline familiarity with appliance energy use and digital tools. Each household then received the EnergiQ hardware kit and a short tutorial delivered in person or via video on how to access and use the platform. Then, using the EnergiQ mobile or web app, participants were guided through a simple and intuitive device pairing process. For each smart plug, the user was prompted to select the appliance it was connected to from a list (e.g., fridge, oven, washing machine). This step enabled accurate device-level tracking without requiring technical configuration.

Once setup was complete, users interacted with the platform as part of their daily routine. EnergiQ monitored appliance-level consumption, detected anomalies, and delivered feedback via natural-language messages generated by the integrated LLM. Each household had at least one active user who received and responded to system feedback. At the end of the deployment period, all participants completed a post-study questionnaire evaluating the clarity, usefulness, and impact of the platform, particularly the LLM-generated recommendations. This structured evaluation allowed us to assess both the technical performance and user experience of EnergiQ under real-world usage.

A summary of the deployment across the 20 apartments is presented in Table 6. Each row corresponds to a participating apartment (Apt) and reports the number of users per household, active users engaging with the platform, and the number of smart plugs deployed. The “Appliances Plugged” column lists the monitored appliances connected through these smart plugs. The “Dur (Ms)” column specifies the number of months of continuous data collection, while “From-To Ms” indicates the exact monitoring period (from October 2024 to March, April, or May 2025). Finally, the “UC” (Use Cases) column shows the specific use cases that were not just designed but actually occurred in practice, were observed, and reported during the monitoring period. These represent real events and user scenarios that took place in the respective households during the experiment.

### 5.2. Performance Evaluation

This section presents a quantitative assessment of EnergiQ’s anomaly detection framework. Reconstruction-based models are evaluated across multiple appliances using standard metrics under varying levels of errors to measure accuracy, robustness, and generalization performance.

#### 5.2.1. Dataset and Evaluation Metrics

Datasets in the literature typically lack detailed information regarding program-specific operations of devices and user-selected programs. They primarily focus on recording power consumption data per operational cycle. In this research, data were gathered from various household appliances, including washing machines, clothes dryers, dishwashers, ovens, water heaters, and refrigerators. These datasets were drawn from a pilot located in Greece.

Each appliance underwent different setups and workloads to ensure comprehensive data capture. Additionally, the classifier models were trained on a dataset consisting of 15,000 labeled appliance consumption sequences drawn from publicly available datasets (REFIT and UK-DALE). The data was split into 80% for training and 20% for testing.

Additionally, simulated datasets were used to simulate anomaly patterns for each device [30]. Three levels of errors (15%, 25%, and 35%) were introduced across all devices, affecting distinct phases of their operation cycles. For washing machines, clothes dryers, ovens, and water heaters errors were simulated during the heating phase, while for dishwashers, errors were simulated during both the washing and drying phases.

For the training phase of the Hybrid Autoencoder anomaly detection method, normal data were used, while data from the simulated dataset were utilized in the testing phase. Additionally, various evaluation metrics were employed to assess the method, including accuracy, recall, precision, and F1-score.

#### 5.2.2. Evaluation of the Device Detection Method

To evaluate the performance of the XGBoost classifier, a comparison with three widely adopted machine learning algorithms, Random Forest, Support Vector Machine (SVM), and K-Nearest Neighbors (KNNs), was conducted. All models were trained and tested on the same dataset consisting of labeled time-series energy signatures representing various appliance types. The results are illustated in Table 7.

The XGBoost model achieved the highest performance across all four metrics. Specifically, it recorded an accuracy of 94% and a precision of 93%, which indicates that when the model predicted a specific appliance, it was correct 93% of the time, minimizing false positives. Additionally, recall value was close to 92%, meaning that the model successfully identified 92% of the true instances for each appliance class, demonstrating strong sensitivity. The F1-score of 92.5% balances these two metrics, confirming that XGBoost maintains a high level of overall classification quality.

In comparison, Random Forest also performed well, achieving an accuracy of 91%, precision of 90%, recall of 89%, and F1-score of 89.5%. While slightly lower than XGBoost, these results still indicate strong classification capabilities. SVM yielded moderate performance with an accuracy of 88% and a precision of 85%. Its recall (86%) and F1-score (85.5%) were slightly better than KNN but showed a decline in generalization to complex appliance patterns. KNN, while straightforward to implement, had the lowest performance in the comparison. It achieved an accuracy of 86%, precision of 83%, recall of 81%, and an F1-score of 82%. This suggests that KNN struggles with overlapping feature distributions and does not scale as effectively with complex appliance signatures. Overall, these results highlight the effectiveness of XGBoost in real-world appliance classification tasks, reinforcing its role as the primary classifier in the EnergiQ system.

#### 5.2.3. Evaluation of the Anomaly Detection Hybrid Autoencoder

To evaluate the effectiveness of the proposed anomaly detection mechanism in EnergiQ, a series of experiments was conducted using both real and simulated data from five household appliances: washing machine, dishwasher, oven, water heater, and refrigerator. The anomaly detection model, based on a hybrid CNN-LSTM autoencoder architecture, was assessed in comparison with three state-of-the-art methods: an LSTM autoencoder, a Simple Autoencoder (AE) using fully connected layers, and a Variational Autoencoder (VAE).

The LSTM autoencoder consisted of two LSTM layers in both encoder and decoder, each with 64 hidden units, trained using the Adam optimizer with a learning rate of 0.001 over 100 epochs. The Simple Autoencoder was built with three dense layers for both encoding and decoding, using ReLU activations and a bottleneck of 32 units. The VAE model employed a symmetrical dense encoder-decoder with Gaussian latent space sampling, optimized to minimize the variational lower bound. All models were trained solely on normal data, and anomaly scores were computed using mean absolute error (MAE).

The comparative average results across all devices are presented in Table 8. These results represent the mean performance values aggregated across all five appliances and averaged over three different levels of injected anomalies: 15%, 25%, and 35%. The hybrid model achieved the best overall performance, with an RMSE of 0.030 and an F1-score of 0.902, outperforming both the LSTM autoencoder (RMSE = 0.038, F1 = 0.857), the AE (RMSE = 0.042, F1 = 0.842), and the VAE (RMSE = 0.041, F1 = 0.843). The improvement is attributed to the hybrid model’s ability to capture both local feature correlations and long-range temporal dependencies.

In addition to the average comparison, the performance of the hybrid model was analyzed across each appliance under different anomaly types. Anomalies were inserted into operational windows such as heating, idle, or load cycles, simulating behavioral drifts, component wear, or misconfigurations. As shown in Table 9, although detection performance decreases slightly as error intensity increases, the hybrid model maintained robust results across all devices.

The washing machine and refrigerator maintained consistently high F1-scores (above 0.90) across all error levels, suggesting that the model effectively captures both cyclical patterns and continuous operational behaviors. The dishwasher and water heater demonstrated similarly strong performance, with only minor degradation observed as error levels increased. This gradual decline in metrics is expected and reflects realistic sensitivity to increased deviation from learned normal behavior.

The oven, being more prone to irregular usage and non-periodic power spikes, exhibited slightly more variability in accuracy and recall. However, even under 35% anomaly error, the F1-score remained within acceptable limits, underscoring the model’s ability to generalize to devices with less predictable patterns. The reconstruction RMSE remained low across all devices, indicating minimal deviation between reconstructed and actual signals under normal conditions, and further validating the model’s reliability in distinguishing anomalous deviations from acceptable variations.

## 6. EnergiQ User Interface and Use Cases

This section describes the practical aspects of user interaction with the EnergiQ platform. It includes an overview of the system’s user interface, design principles, and key functionalities across web and mobile applications. Real-world use cases are also presented to illustrate how users engage with the platform to monitor, interpret, and optimize appliance-level energy consumption.

### 6.1. User Interface

#### 6.1.1. Main Menu

**Figure 5:**
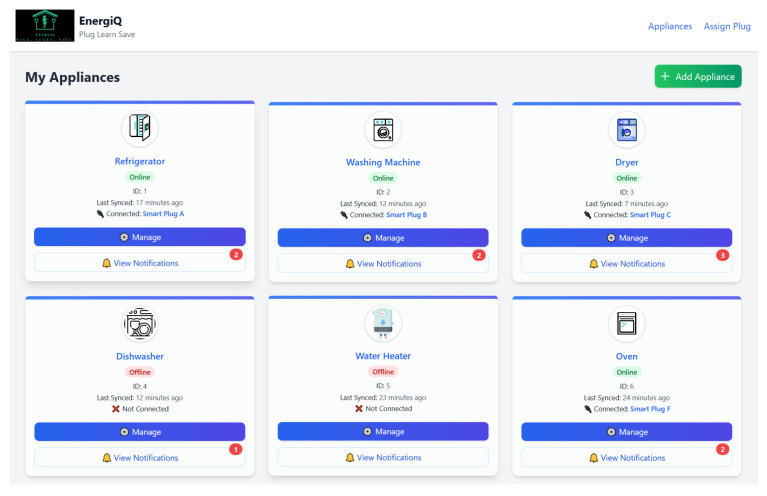
Main dashboard displaying all registered appliances with status indicators and quick access to controls.

The principal screen of the EnergiQ, represented in Figure 5, enumerates all the registered devices in the form of cards. Each of these cards shows vital details like device name, status (online/offline), ID, last sync date, and corresponding smart plug. This arrangement makes it easy for users to obtain an immediately visible overview of their home appliances and their states of operation. In addition, the platform is very responsive, ideal for mobile screens through a vertically organized format, as presented in Figure A2. This makes tracking and monitoring the appliances much easier for users from both desktop and mobile platforms.

**Figure A1:**
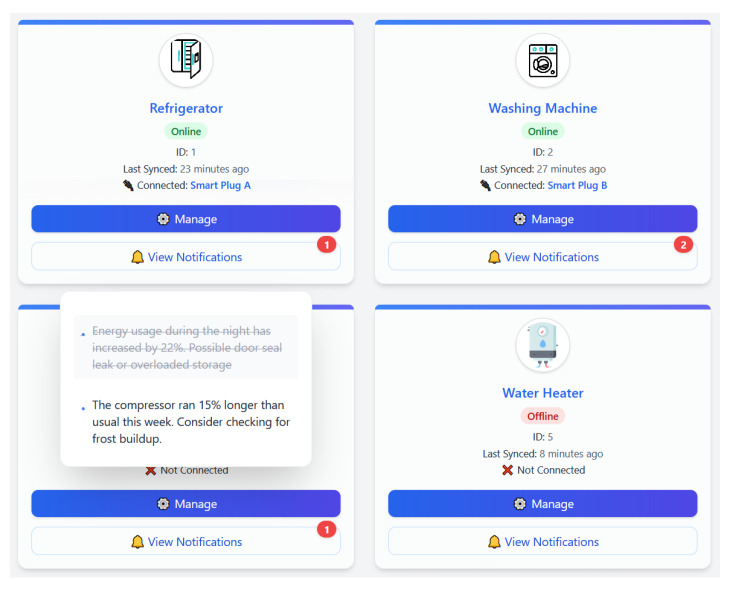
Notification panel showing natural language alerts for unusual appliance behavior.

**Figure A2:**
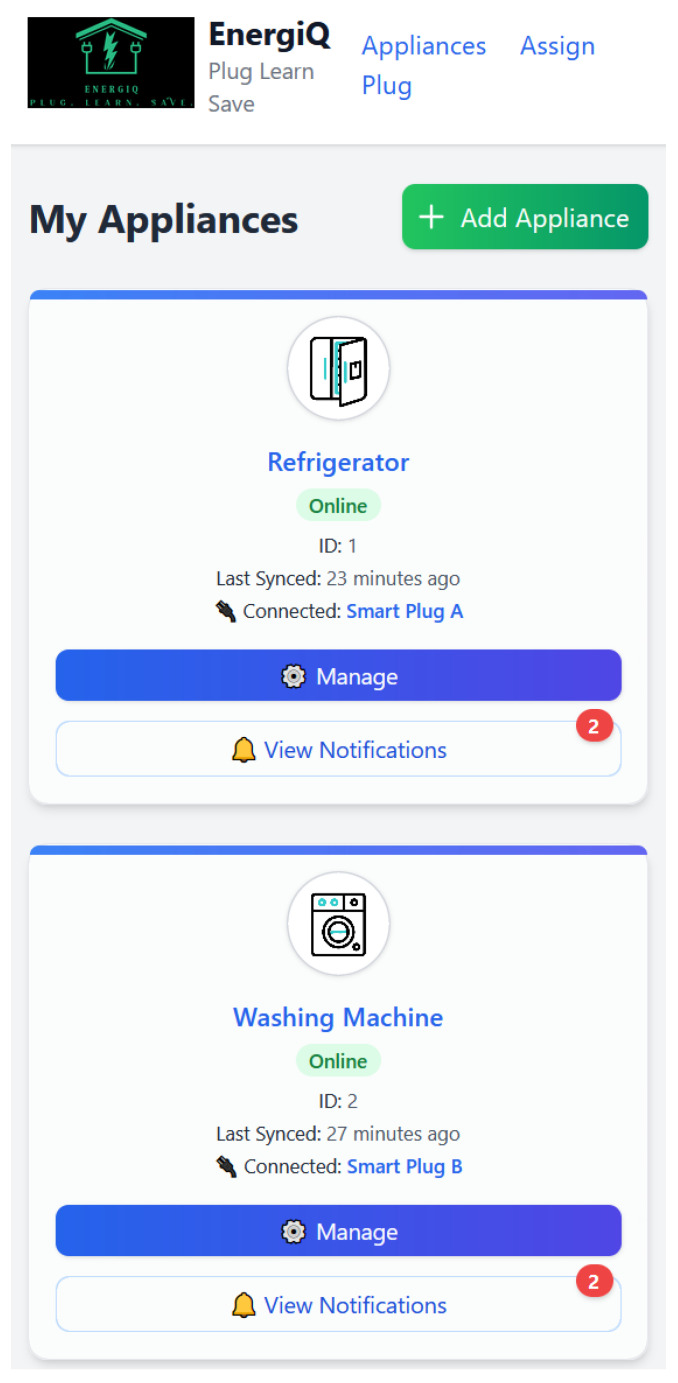
Main surface application, mobile view.

The notification system serves as the cornerstone of user awareness. When the “View Notifications” button is selected, users are presented with natural language alerts highlighting unusual activities, such as prolonged usage or deviations from typical patterns, as illustrated in Figure A1. For further analysis, users can click the “Manage” on the card of any device. An elaborate appliance view is presented in Figure A4, where users can analyze energy usage patterns, review historical warnings, and identify potential issues.

#### 6.1.2. Use Case 1: Normal Appliance Operation

**Figure A3:**
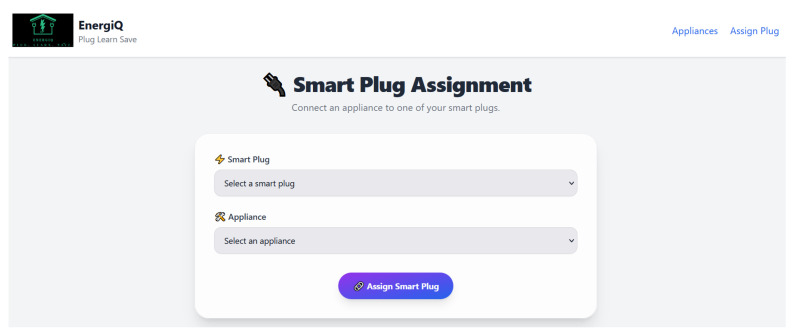
Interface for assigning a smart plug to an appliance using simple drop-down selections.

**Figure A4:**
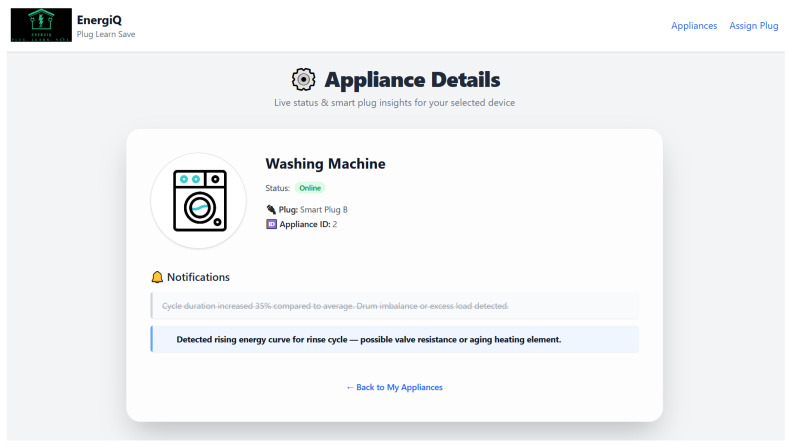
UI of appliance.

This use case illustrates the use of the EnergiQ platform to identify household appliances in normal, fault-free use. The procedure starts with the user assigning a smart plug to an appliance by using the intuitive EnergiQ interface, shown in Figure A3. Two drop-down menus are provided on the interface, one to choose an available smart plug and one to choose the related appliance type. Upon confirmation with the “Assign Smart Plug” button, the mapping between the appliance and the energy stream was established. In this instance, the refrigerator is connected to smart plug A, the washing machine to smart plug B, the dryer to smart plug C, the dishwasher to smart plug D, and the water heater to smart plug E. This is an important step, as it associates incoming energy data with an appliance name, facilitating proper monitoring and analysis to occur. The simple structure allows for non-technical users to do the procedure easily and with haste, allowing for onboarding without issues and universal adoption.

When the task is finished, the appliance starts transmitting real-time telemetry voltage, current, and power to the EnergiQ platform. The machine learning engine repeatedly monitors the data and verifies that the pattern of usage is in accordance with learned past behavior. In this case, the platform registered zero departures from previously established behavioral patterns. Thus, the system interface showed a green status icon to reflect normal functioning. No alerts are sent, maintaining the user experience non-intrusive. In the background, the system silently records the usage data to continue to improve the appliance’s behavioral model. This ongoing learning improves the platform to pick up on subtle patterns in future usage.

#### 6.1.3. Use Case 2: Device Replaced Without User Notification

This use case implies that the EnergiQ platform is able to independently identify changes in appliance usage patterns, implying a device has been replaced without user indication. Since they have paired initially, EnergiQ tracks each appliance’s energy consumption profile as observed through statistical and temporal features. In this scenario, the user physically replaces the washing machine connected to smart plug B with a new one, without informing the system. When the new appliance is plugged into the home smart plug, the incoming power data starts to diverge substantially from the consumption patterns evolved so far. The machine learning module detects this divergence in behavior through classification and anomaly detection models and flags it as inconsistent with the historical trend.

**Figure 6:**
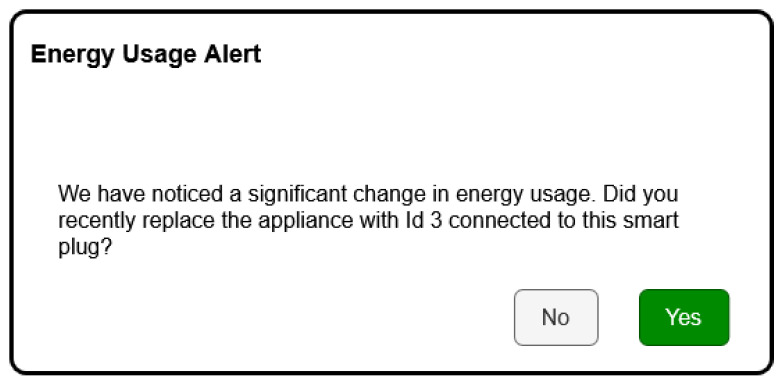
Alert prompting the user to confirm a detected appliance replacement.

Rather than producing a technical error message, the LLM reasoning layer transforms the anomaly into a natural language warning, as can be seen in Figure 6. This notification is received through the web or mobile interface and asks the user to confirm or correct the appliance identity of the associated smart plug. The user responds with “Yes” and is then redirected to the appliance assignment interface (see Figure A3), where the user can correctly reallocate the smart plug to the correct appliance. This process allows the platform to correctly update its internal models, so accurate monitoring continues with low-user-effort requirements. The successful handling of this scenario is a testament to EnergiQ’s strength when coping with unexpected hardware modification without data loss and delivering a seamless, low-friction user experience.

#### 6.1.4. Use Case 3: Abnormal Consumption Suggests Fault

This is an illustration of the way that the EnergiQ system can detect anomalous use patterns that could be indicating an imminent failure in an appliance. In this scenario, the refrigerator connected to smart plug A was still operational but began to deviate from its established usage profile exhibiting prolonged cycles. The CNN-LSTM-based anomaly detection unit identified this divergence through its comparison of real-time telemetry with the learned normal pattern. The system marked the activity as anomalous when the reconstruction error was above a threshold.

**Figure 7:**
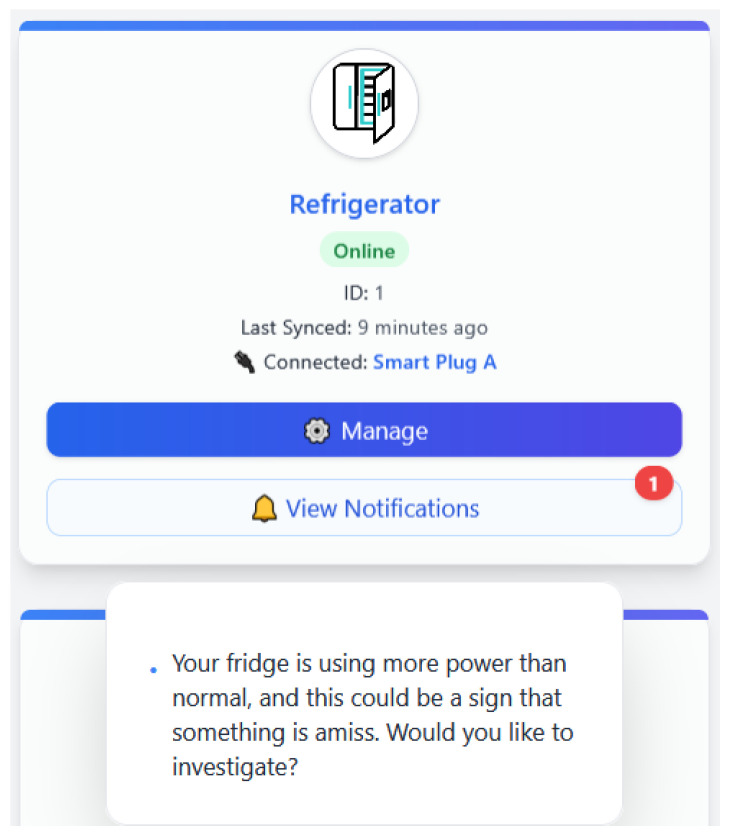
Alert indicating abnormal energy consumption by a refrigerator, prompting the user to investigate potential issues.

The LLM reasoning layer then maps the technical anomaly to a natural language alert; for example, in Figure 7. Instead of inundating the user with extensive diagnostic information, the system presents a compact, actionable message, indicating a suspected problem and invoking user inspection. This result demonstrates how EnergiQ enables proactive maintenance through early detection of inefficiencies, avoidance of failure, and prolongation of appliance life, while improving energy efficiency overall.

#### 6.1.5. Use Case 4: Unauthorized or High-Load Appliance Detected

**Figure 8:**
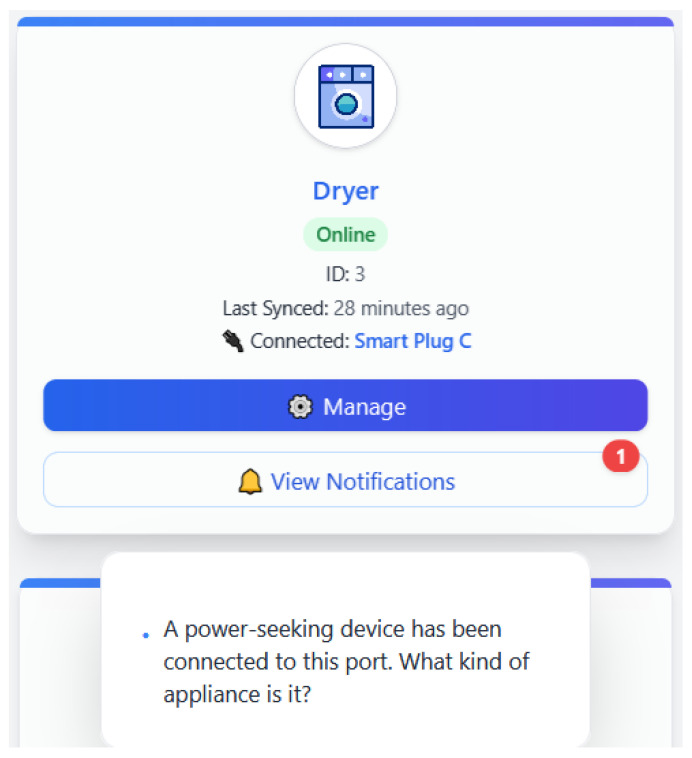
Notification alerting the user to an unrecognized high-power device, prompting appliance identification.

This use case illustrates EnergiQ’s capability to identify an unplugging of a surprising plug-in or extremely energy-consuming appliance into a smart plug. In this scenario, the dryer connected to smart plug C was plugged in without prior registration, causing a sudden and high-magnitude spike in energy consumption. The platform identifies such a rare and high-magnitude energy consumption pattern with no connection to any of the pre-introduced appliance profiles. The machine learning algorithm reports this out-of-the-ordinary activity as a red flag, and LLM reasoning layer creates a natural language alert, which appears as the message seen in Figure 8. The notification warns the user about the heavy-load operation and asks them to categorize the appliance.

This alarm system in advance prevents the unwanted or dangerous use from going unnoticed. The user can identify the device, turn off the alarm, or switch off the appliance if needed. This application demonstrates EnergiQ’s alertness in monitoring energy activity and its capability to keep an eye on household energy consumption and avoid the dangers of runaway energy bills or electrical overloads.

#### 6.1.6. Use Case 5: Energy Optimization Suggestion Based on Usage Patterns

This use case application illustrates EnergiQ’s ability to facilitate energy efficiency through behavioral pattern detection and recommending cost-saving behavior. In this scenario, the dishwasher connected to smart plug D was operating during peak evening hours (6–9 p.m.), when electricity tariffs are typically higher. While the usage behavior is typical and there is no deviation, the system identified an opportunity for cost and energy savings based on timing.

**Figure 9:**
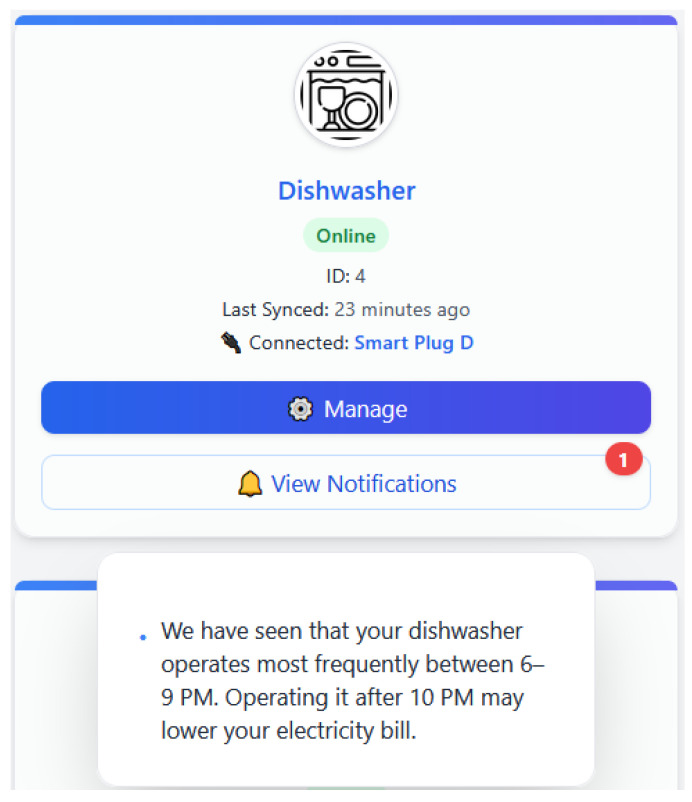
Recommendation suggesting off-peak operation of the dishwasher to optimize energy costs.

The use times are computed by machine learning models, and the LLM reasoning engine outputs a natural language recommendation, as shown in Figure 9. The recommendation is displayed non-intrusively on the user interface, prompting the user to consider changing appliance use to off-peak periods.

#### 6.1.7. Use Case 6: Appliance Left on Unexpectedly

This use case illustrates how EnergiQ can identify excessively long durations of appliance on times that signal neglect, wastage of energy, or even safety hazards. Here, the water heater attached to smart plug E was found to have been operating non-stop for an extremely long time outside of its typical usage behavior. The machine learning engine compared the uptime to learned historical activity and marked the event as suspicious based on its duration.

**Figure 10:**
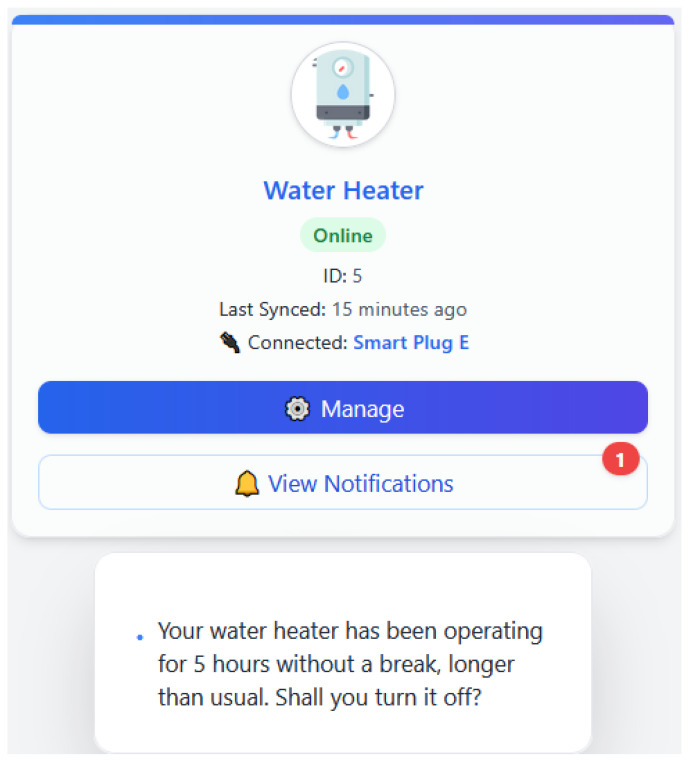
Notification warning the user about prolonged operation of a water heater and suggesting manual intervention.

The LLM module produces a usage-duration natural language warning, like the one presented in Figure 10, warning the user that the appliance has been used for a greater duration than usual. The warning is presented via the user interface, normally accompanied by the possibility of taking action immediately. This is just one way that EnergiQ ensures home safety and saves energy through continuous monitoring of usage patterns and reminding the user of possible risky or undeserved circumstances.

### 6.2. Scalability and Replicability

EnergiQ is based on a modular, service-oriented architecture that facilitates scalable deployment in multiple residential settings and is highly replicable with little configuration. Plug-and-play IoT installation involving smart plugs and edge gateways such as Raspberry Pi makes it easy to install and quickly adopt without any technical support, making deployment feasible in small- and large-scale home networks. The application of MQTT for low-latency communication guarantees efficient data transfer in a reliable manner, even under bandwidth-limited scenarios. The backend software, which was implemented using the Laravel framework and Redis-run asynchronous queues, allows concurrent processing of telemetry data as well as scalable ML inference operations. This renders the system resilient under rising data volumes and user bases.

Most importantly, EnergiQ’s time-series analytics and feedback creation are time zone agnostic. All the energy profiling, anomaly detection, and LLM-based recommendation functions function relative to local device time and are independent of any geographic or temporal setting. This allows the platform to perform uniformly regardless of region without needing synchronization or localization setups. In addition, the ML and LLM components of the platform are built as separate microservices that can be duplicated and horizontally scaled. The retraining pipeline that is feedback-driven guarantees that the system learns from real appliance behavior over time, while maintaining its generalizability and improving its accuracy. The LLM reasoning layer, bolstered by instruction-tuned models and Retrieval-Augmented Generation (RAG), provides consistency and clarity in communication between diverse appliance types and user preferences.

In practice, the physical size of a household does not directly impact the operation of EnergiQ, as the number of core appliances typically remains within a similar range, even in larger homes. The system’s modular plug-and-play IoT layer easily supports the addition of extra smart plugs where needed, without requiring reconfiguration. Moreover, the number of monitored appliances, whether small or large, does not affect the performance or scalability of EnergiQ’s analytics pipeline, as each device operates independently within the system’s architecture. Regarding varied energy consumption profiles, the platform inherently accommodates diversity through its personalized feedback mechanisms. EnergiQ’s LLM reasoning and anomaly detection are specifically tailored to each user’s household behavior, meaning the system adapts to unique consumption patterns rather than relying on generalized models. This ensures that variability in appliance types, user routines, or energy profiles does not compromise the accuracy or relevance of recommendations provided.

### 6.3. User Evaluation of ENERGiQ

A core component of EnergiQ is its LLM-powered messaging layer, which transforms complex energy data into clear, user-friendly feedback. This subsection examines how these recommendations influenced user understanding, trust, and behavior during the deployment period.

**Figure A5:**
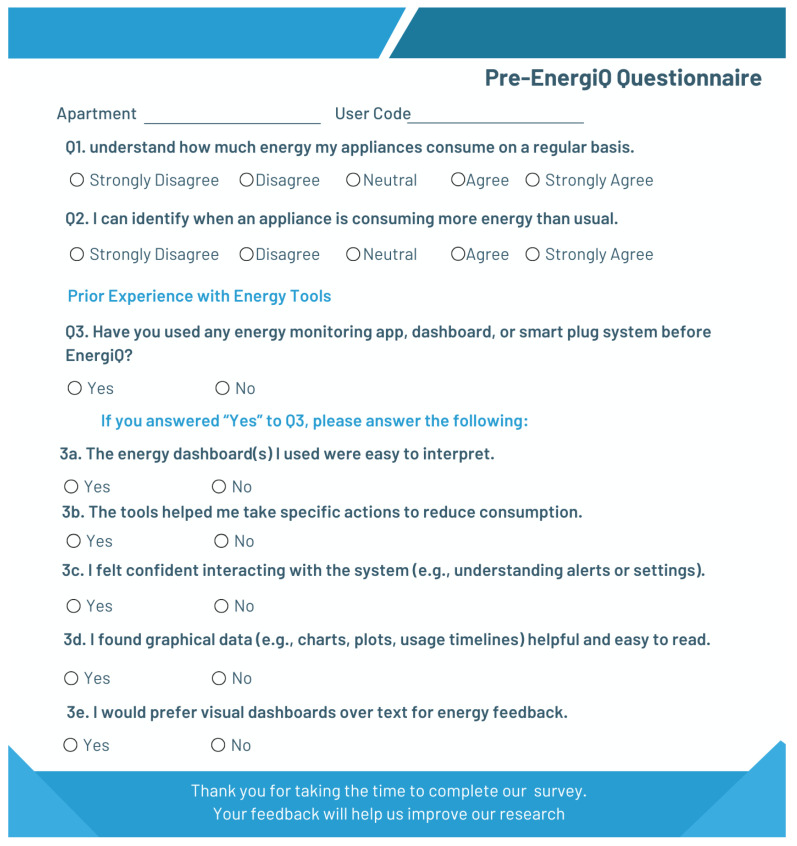
Pre-EnergiQ user questionnaire.

To evaluate the added value of LLM-generated feedback, a pre/post questionnaire with participants before and after using EnergiQ for a period of 6–8 months was conducted, as discussed in Section 5.1. The aim was to assess perceived clarity, usefulness, and trust in energy-related information, comparing conventional dashboards with LLM-enhanced messaging. Participants (n = 38 active users) across 20 apartments were asked to rate their agreement with the following statements on a 5-point Likert scale (1: strongly disagree to 5: strongly agree). The questionnaire was conducted in two stages: prior to deployment (to assess baseline awareness and habits) and after the test period (to evaluate the usability and impact of the EnergiQ platform). The Pre-EnergiQ questions are as depicted in Figure A5.

The pre-deployment questionnaire was designed to assess participants’ baseline perceptions and experiences related to energy consumption awareness. Q1 aimed to capture users’ perceived understanding of their appliance energy consumption, while Q2 assessed whether users felt capable of identifying irregular or excessive consumption patterns. Q3 explored prior experience with energy-monitoring tools, recognizing that users familiar with such technologies may have different expectations or awareness levels. The follow-up questions (Q3a to Q3e) specifically targeted users with prior experience to evaluate how effective existing platforms were in supporting their energy-related decisions (e.g., through dashboards, charts, or alerts). These questions can help identify potential gaps in usability and comprehension in current solutions gaps, which EnergiQ aims to address with its more intuitive, LLM-based feedback approach.

**Figure 11:**
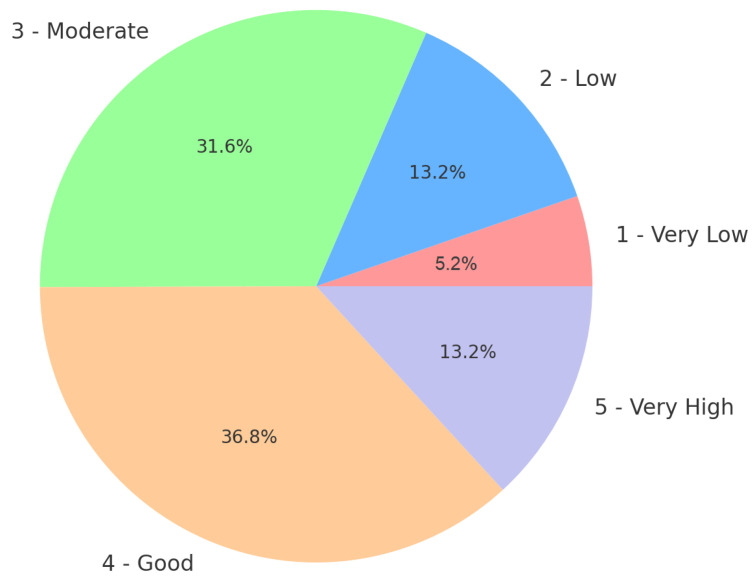
Participants’ self-assessed understanding of appliance energy consumption.

The results of the pre-deployment questionnaire, summarized in Table A1, provide insight into participants’ baseline understanding of appliance energy consumption and their prior experience with energy-monitoring tools. Overall, users reported moderate self-assessed awareness. Specifically, the participants rated their understanding of how much energy their appliances consume on an average of 3.55 out of 5 Figure 11, and their ability to detect unusual energy use at 3.50 Figure A7.

However, this likely reflects perceived rather than actual capability, as most participants did not have access to detailed monitoring tools prior to using EnergiQ Figure A8. This points to a gap between awareness and actionable insight, reinforcing the need for interpretable feedback mechanisms. Regarding prior tool usage, 42% (16 out of 38) of respondents indicated having used some form of energy-monitoring system before (Q3 = Yes). Among these users, the feedback on past tools was mixed. The average rating for ease of interpretation was 3.44, the ability to take action based on feedback scored 3.31, the confidence in using these systems was 3.44, and the perceived helpfulness of charts and visual data averaged 3.19.

These results indicate that even for users familiar with monitoring tools, the perceived usefulness and usability of existing solutions were limited. Notably, when asked whether they preferred visual dashboards over text-based guidance, only 8 out of 38 users responded affirmatively, while 14 expressed a preference for alternatives. This highlights the relevance of EnergiQ’s approach, which emphasizes natural language guidance generated through LLMs to bridge the gap between data and actionable understanding. These findings support the rationale for EnergiQ’s design, which focuses not merely on providing detailed energy data but on delivering insights in a user-friendly, interpretable format that promotes informed decision-making.

**Figure A6:**
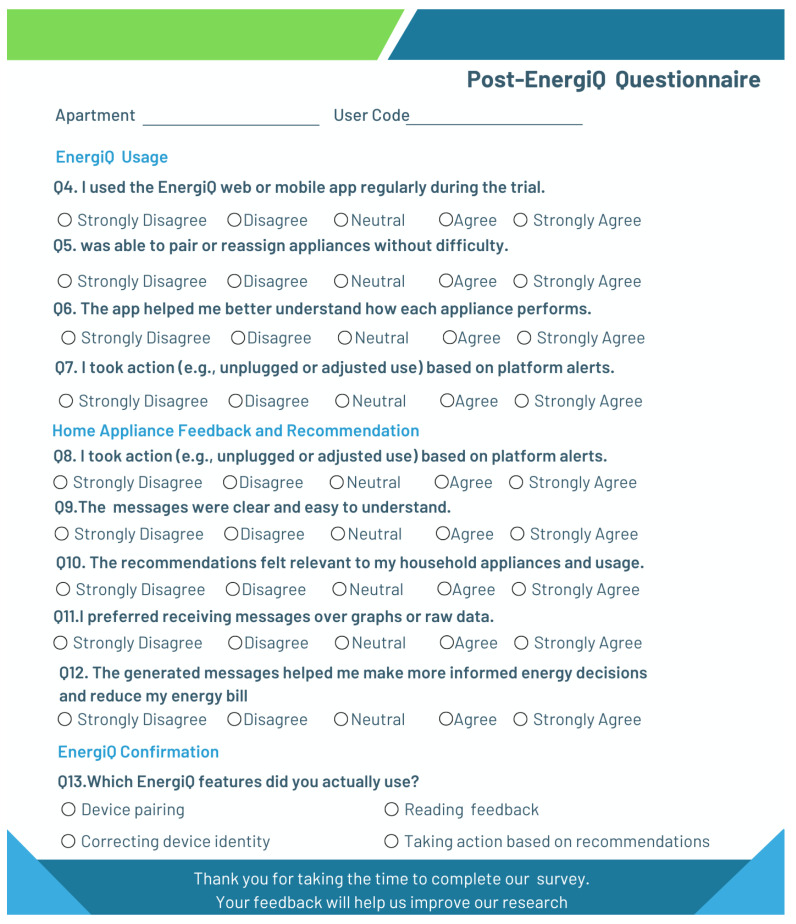
Post-EnergiQ user questionnaire.

**Figure A7:**
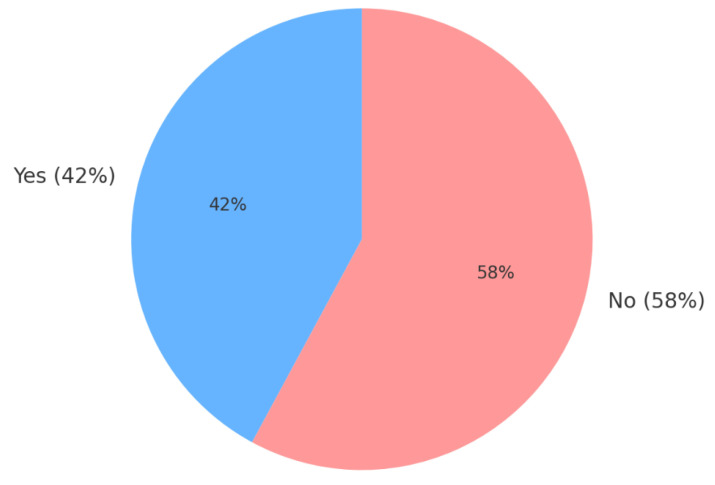
Prior use of energy-monitoring systems (Q3).

**Figure A8:**
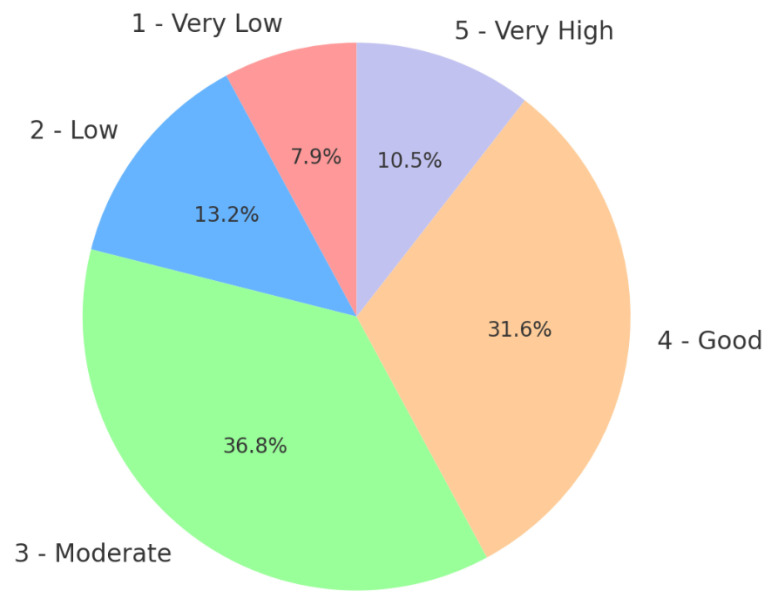
Q2: Self-assessed ability to detect unusual energy use.

The post-deployment questionnaire was completed by 38 active users who engaged with the EnergiQ platform. It evaluates app usability, user interaction with system recommendations, and the clarity and impact of LLM-generated feedback. Users were also asked to confirm which EnergiQ features they actually used during the study, including device pairing, feedback reading, and taking energy-saving actions. The Post-EnergiQ Questions are as depicted in Figure A6. It should be noted that EnergiQ provides appliance-specific recommendations directly, without requiring users to interpret consumption patterns themselves. Therefore, user responses reflect their general comprehension of the feedback rather than of any specific appliance behavior. As this work focuses on validating the EnergiQ methodology rather than delivering a comprehensive user behavior study, the questionnaire was intentionally kept concise; a more detailed user study would be considered in future, market-ready iterations. Q13 also included ‘taking action based on recommendations’ as a response option to capture whether users reported acting upon EnergiQ’s feedback, which is considered a key aspect of evaluating the platform’s effectiveness.

**Figure 12:**
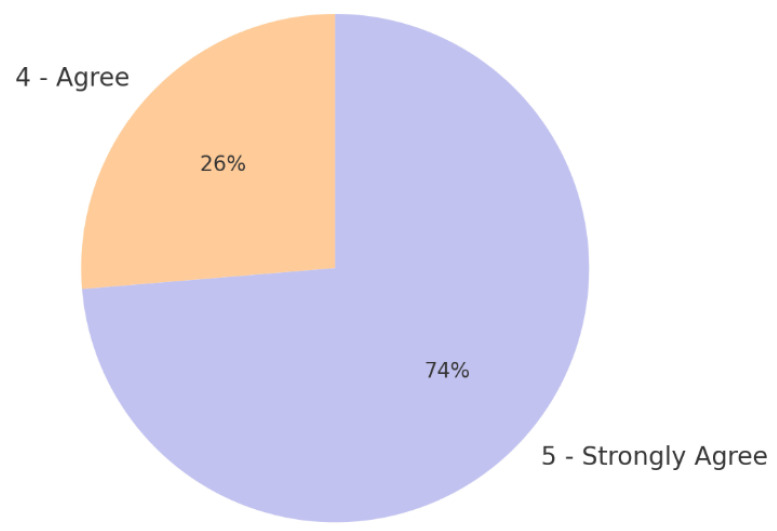
Q4: easy to understand the EnergiQ feedback.

**Figure A9:**
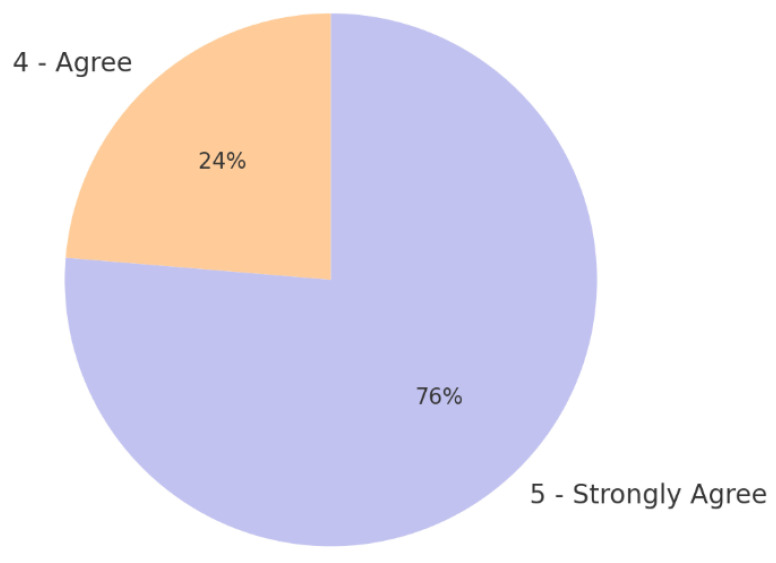
Q5: Satisfaction with EnergiQ feedback.

As summarized in Table A2, users reported consistently positive experiences with the EnergiQ platform across a variety of functional and interpretive dimensions. Participants strongly agreed that the system was easy to use, with device pairing (Q5) scoring an average of 4.79 Figure A9, and routine app usage (Q4) close behind at 4.61 Figure 12, confirming the accessibility and low-friction design of the user interface.

The LLM-generated natural language feedback was also highly rated. Users gave an average score of 4.63 for message clarity (Q9) and 4.74 for the contextual relevance of recommendations (Q10), indicating that the system’s feedback was both understandable and personalized to household needs. Notably, 66% of users agreed or strongly agreed (Q12) that the platform helped them make more informed energy decisions and contributed to reducing their energy bills.

**Figure A10:**
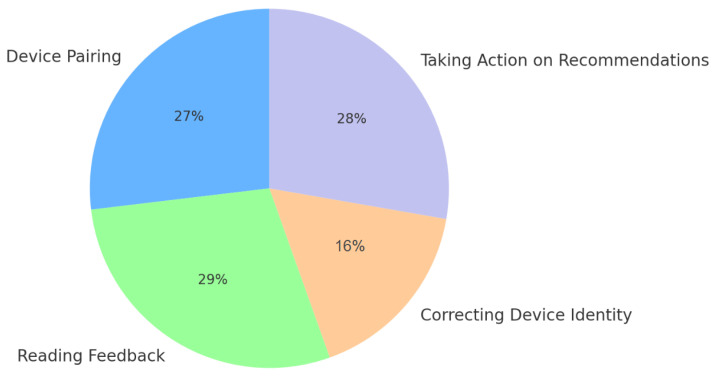
Q13: Features users engaged with EnergiQ.

Regarding the overall impact of the platform, users acknowledged improved appliance-level understanding (Q6: 4.61) and behavioral adjustments prompted by alerts (Q7: 4.39). The preference for textual feedback over visual dashboards (Q11: 4.79) was consistent with pre-study trends, reinforcing the benefit of language-based interfaces for non-technical audiences. Finally, the platform’s feature usage profile (Q13) showed high engagement: the most commonly used features were device pairing, reading feedback, and taking action based on system recommendations, all of which were selected by a majority of participants (Figure A10). This highlights EnergiQ’s effectiveness not only in delivering insights but in enabling users to act on them, supporting meaningful behavior change over the 6–8 month trial period.

In order to measure real-world effectiveness and usability of EnergiQ, we administered a post-deployment user satisfaction survey to the 20 participating homes in our 6–8 month field trial. Results indicated that 87% of users rated the system as intuitive and easy to use, with specific praise for plug-and-play convenience and simplicity of the interface. Most significantly, 90% of the participants indicated that natural-language recommendations from the LLM could be easily understood without learning technical skills. Users reported that such recommendations were “personalized,” “actionable,” and “easy to trust,” and resulted in more informed decisions about appliance utilization. In addition, more than 70% of the participants attested to having changed at least one energy-related habit based on the recommendations provided. These results emphasize the potential of the platform to translate intricate energy analytics into actionable suggestions, which prove the efficacy and usability of the system on household energy habits.

## 7. Conclusions

This paper presented EnergiQ, a smart, LLM-powered platform that translates and fine-tunes appliance-level home energy usage. By integrating IoT-based smart sensing, statistical and deep models, and natural language reasoning with LLMs, EnergiQ closes the gap between intricate energy information and understandable, actionable data. The platform not only properly classifies appliances and identifies anomalies but also provides an active feedback loop that continuously improves its performance through user feedback. When deployed in real-world operation in 20 homes, the system was technically stable and intuitive, enabling non-technical users to interact with their energy consumption in a meaningful manner.

EnergiQ’s natural language-based recommendation integration, horizontally deployable architecture, and modular design present it as a strong contender to drive next-generation home energy management. Application of LLMs revolutionizes traditional energy feedback systems by enabling context-aware, behavior-guided recommendations that elicit anticipatory and sustainable behavior.

Next steps can include extending coverage to a greater diversity of appliances, improving fault prediction, and adding more profound levels of personalization. Although the present emphasis continues to be on appliance-level monitoring compared to more general home control or automation (such as HVAC or multi-purpose energy-saving technologies), we do see potential for eventual convergence with outside home automation systems, which would also serve to enhance EnergiQ’s flexibility and scale. While EnergiQ showcases the capability of LLMs to encourage consumers to adopt energy-saving behavior, we recognize model inference holds a marginal computational expense. Yet, with light-weight instruction-adapted models and local caching techniques, the platform reduces additional carbon emissions while maintaining the environmental cost of reasoning to be zero compared to saved energy. In general, EnergiQ provides a solid foundation for more adaptive, transparent, and user-centric energy systems that enable homes to become active participants in the shift towards smarter and cleaner energy use.

## Figures and Tables

**Table 1:** Overview of traditional and advanced platforms in home automation and battery management.

Platform/System	Type	Features	Openness	Citation
Home Assistant	Smart Home SPA	Modular voice-based assistant with IoT integration, NLP, and blockchain support. Community-driven and customizable.	Open source	[24]
NRG4-U	Home Energy Management System (HEMS)	Non-intrusive ML-driven tool using minimal data to generate unique comfort/load profiles, and personalized energy-saving recommendations.	Semi-open (custom system, not fully open source)	[25]
Conventional BMS	Battery Management System	Supports cell balancing, thermal regulation, state estimation, and basic safety management. Limited scalability and adaptability.	Closed	[26]
Intelligent BMS (IBMS)	Advanced BMS (End-Edge-Cloud)	Integrates digital twins, AI, blockchain, IoT, and multi-layer computing for advanced diagnostics, predictive maintenance, and real-time control.	Closed	[26]

**Table 2:** Summary of research gaps in NILM, ML, and LLMs for smart energy systems.

Reference	Identified Research Gap	Proposed Solution
[11]	Limited generalization and model portability of NILM algorithms across diverse household settings.	Development of adaptive, transfer learning-based NILM models to enhance performance across heterogeneous environments.
[12]	Lack of standardized evaluation metrics and reproducibility in NILM research.	Introduction of NILMTK benchmarking toolkit to enable fair comparisons and foster reproducibility.
[13]	Difficulty in detecting low-power appliances due to low sampling rates.	Incorporation of higher-frequency data acquisition and hybrid signal-deep learning models for improved granularity.
[14]	Limited forecasting accuracy in traditional NILM methods.	Use of LSTM and GRU-based models to enhance time-series forecasting and appliance disaggregation accuracy.
[16]	Traditional ML approaches underperform with high-dimensional, dynamic energy data.	Shift to deep learning models (RNNs, LSTMs) to better capture sequential and temporal dependencies.
[17]	Insufficient appliance coverage and real-world testing in NILM systems.	LSTM-based multi-meter method supports identification of up to 16 appliances with competitive results.
[18]	Underutilization of ML in critical areas like theft detection and renewable forecasting in developing countries.	Expansion of AI/ML in energy equity, maintenance prediction, and theft mitigation for developing regions.
[19]	Lack of standardized datasets and challenges in cross-environment NILM deployment.	Emphasis on public datasets and robust DL models (e.g., multiscale residual networks) to improve generalizability.
[20]	Manual building energy modeling is time-consuming and expertise-dependent.	Eplus-LLM enables natural language to EnergyPlus model translation, reducing modeling time by over 95%.
[21]	Lack of formal design and explainability in LLM-based energy tools.	Graph-theoretic LLM modeling frameworks enhance interpretability and performance in smart energy systems.

**Table 3:** Summary of extracted statistical features.

Feature Name	Purpose
Mean Power	Captures overall energy usage
Standard Deviation	Measures consumption variability
Peak-to-Average Ratio (PAR)	Highlights power spikes
Rolling Mean	Identifies gradual trends
Rolling Standard Deviation	Detects local fluctuations
Entropy	Measures signal unpredictability
Skewness	Identifies load asymmetry
Kurtosis	Detects sharp power peaks
Proportion Above Threshold	Distinguishes on-time behavior
Periodogram Peaks	Captures periodic usage patterns
Matrix Profile	Finds repeated usage cycles

**Table 4:** Energy-saving recommendations for various household devices.

Recommendation	Input_Real	Frequency
Avoid using high-temperature cycles unnecessarily; heating water is the biggest energy load.	Device: Washing Machine Error: More Duration	High
Turn off the machine after use; some models consume standby power even when not in use.	Device: Dishwasher Error: High Idle Consumption	High
Use lower heat settings for lightly soiled or smaller loads to reduce peak power draw.	Device: Dryer Error: Excessive Power Usage, General Usage	Low
Run a self-cleaning cycle only when truly necessary; it draws significant power and wears components.	Device: Oven Error: Excessive Self-Cleaning, General Usage	Low
If heating is slow or inconsistent, check for an inefficient gas valve or heating element.	Device: Water Heater Error: Inefficient Gas Valve or Heating Element, Major	Low
Avoid leaving the door open for extended periods.	Device: Fridge Error: Door Open	High

**Table 5:** Overview of the recommendation dataset.

Column Name	Description	Non-Null Entries
No	Entry number or index	100
Output (Recommendation)	Final user recommendation text	100
For Us (Inferred Pattern)	Pattern inferred from energy consumption data	100
Input1 (Real Input)	Real-world trigger inputs (e.g., device error messages)	40
Input2 (Frequency)	Frequency label (e.g., High, Medium, Low)	40

**Table 6:** Summary of experimental setup across 20 apartments for the 36.

Apt	Users per Apt	Active Users	Smart Plugs	Appliances Plugged	Dur (Ms)	From-To Ms	UC
1	2	2	4	Washing Machine, AC, Water Heater, Oven	6	10/24–3/25	1, 5
2	3	3	2	Dishwasher, Washing Machine	7	10/24–4/25	1, 2, 5
3	2	2	3	Oven, Washing Machine, Dryer	8	10/24–5/25	1, 3, 4, 5, 6
4	4	2	2	Water Heater, Dishwasher	8	10/24–5/25	1, 3, 4, 5, 6
5	4	2	2	Oven, Dishwasher	7	10/24–4/25	1, 2, 5
6	2	1	3	Water Heater, Washing Machine, AC	6	10/24–3/25	1, 5
7	2	2	2	Dryer, Washing Machine	7	10/24–4/25	1, 2, 5
8	3	2	4	Oven, AC, Fridge, Dryer	8	10/24–5/25	1, 3, 4, 5, 6
9	3	1	4	Dishwasher, AC, Washing Machine, Oven	8	10/24- 5/25	1, 3, 4, 5, 6
10	2	1	2	Fridge, Oven	7	10/24–4/25	1, 2, 5
11	3	2	4	AC, Water Heater, Oven, Dryer	8	10/24–5/25	1, 3, 4, 5, 6
12	4	3	3	Fridge, Dishwasher, Washing Machine	8	10/24–5/25	1, 3, 4, 5, 6
13	3	2	4	AC, Dryer, Fridge, Oven	8	10/24–5/25	1, 3, 4, 5, 6
14	4	2	3	Washing Machine, Oven, Fridge	8	10/24–5/25	1, 3, 4, 5, 6
15	2	2	4	Water Heater, Oven, Dishwasher, Fridge	8	10/24–5/25	1, 3, 4, 5, 6
16	3	1	2	Washing Machine, AC	7	10/24–5/25	1, 2, 5
17	4	3	3	AC, Fridge, Water Heater	8	10/24–5/25	1, 3, 4, 5, 6
18	2	2	4	Fridge, Dryer, Dishwasher, Washing Machine	8	10/24–5/25	1, 3, 4, 5, 6
19	3	1	2	Oven, Fridge	7	10/24–4/25	1, 2, 5
20	2	2	3	Washing Machine, AC, Oven	6	10/24–3/25	1, 5

**Table 7:** Comparison of XGBoost with other classifiers for appliance identification.

Model	Accuracy	Precision	Recall	F1-Score
XGBoost (EnergiQ)	0.94	0.93	0.92	0.925
Random Forest	0.91	0.90	0.89	0.895
Support Vector Machine (SVM)	0.88	0.85	0.86	0.855
K-Nearest Neighbors (KNNs)	0.86	0.83	0.81	0.820

**Table 8:** Comparative performance of reconstruction-based anomaly detection methods.

Model	RMSE	Accuracy	Precision	Recall	F1-Score
CNN-LSTM Hybrid AE	0.030	0.914	0.906	0.898	0.902
AE	0.042	0.860	0.850	0.835	0.842
LSTM AE	0.038	0.875	0.861	0.854	0.857
VAE	0.041	0.868	0.848	0.838	0.843

**Table 9:** Device-level performance of the hybrid autoencoder across different error levels.

Device	Error Level	RMSE	Accuracy	Precision	Recall	F1-Score
Refrigerator	15%	0.024	0.94	0.94	0.932	0.937
	25%	0.022	0.943	0.942	0.927	0.933
	35%	0.028	0.94	0.944	0.934	0.929
Washing Machine	15%	0.032	0.917	0.09	0.898	0.906
	25%	0.036	0.916	0.909	0.9	0.903
	35%	0.033	0.914	0.905	0.903	0.903
Dishwasher	15%	0.031	0.908	0.899	0.89	0.894
	25%	0.033	0.911	0.899	0.893	0.899
	35%	0.033	0.911	0.901	0.885	0.895
Water Heater	15%	0.034	0.9	0.89	0.879	0.882
	25%	0.027	0.897	0.887	0.883	0.883
	35%	0.026	0.894	0.886	0.883	0.883
Oven	15%	0.036	0.877	0.868	0.859	0.864
	25%	0.033	0.88	0.872	0.857	0.868
	35%	0.037	0.885	0.873	0.858	0.866

## Data Availability

The data presented in this study are available on request from the corresponding author.

## Appendix A

**Table A1:** Pre-EnergiQ questionnaire results from the 38 active users.

Q1	Q2	Q3	Q3a	Q3b	Q3c	Q3d	Q3e
4	2	No	No	No	No	No	No
5	4	Yes	Yes	Yes	No	Yes	No
2	4	Yes	Yes	Yes	Yes	Yes	Yes
4	4	Yes	Yes	Yes	Yes	Yes	Yes
4	3	Yes	Yes	Yes	Yes	Yes	Yes
5	5	No	No	No	No	No	No
2	5	Yes	Yes	Yes	Yes	Yes	Yes
2	5	No	No	No	No	No	No
4	5	Yes	Yes	Yes	Yes	Yes	Yes
3	4	Yes	Yes	Yes	Yes	Yes	No
4	3	Yes	Yes	Yes	Yes	Yes	No
4	3	Yes	Yes	Yes	Yes	Yes	No
4	4	No	No	No	No	No	No
4	3	No	No	No	No	No	No
5	4	No	No	No	No	No	No
2	5	No	No	No	No	No	No
5	4	No	No	No	No	No	No
5	5	No	No	No	No	No	No
5	5	Yes	Yes	Yes	No	Yes	Yes
4	2	Yes	Yes	Yes	Yes	Yes	Yes
3	4	Yes	Yes	Yes	No	No	Yes
2	2	Yes	Yes	Yes	Yes	No	Yes
3	4	Yes	Yes	Yes	No	No	No
5	4	Yes	Yes	Yes	No	Yes	No
5	2	No	No	No	No	No	No
3	2	Yes	Yes	Yes	No	Yes	Yes
3	4	Yes	Yes	Yes	No	No	No
3	3	Yes	Yes	Yes	Yes	Yes	No
5	5	Yes	Yes	Yes	Yes	Yes	Yes
5	2	No	No	No	No	No	No
2	5	Yes	Yes	Yes	Yes	Yes	Yes
2	3	No	No	No	No	No	No
5	3	No	No	No	No	No	No
3	3	Yes	Yes	Yes	No	Yes	No
3	2	Yes	Yes	Yes	No	No	Yes
2	3	No	No	No	No	No	No
5	2	No	No	No	No	No	No
2	3	No	No	No	No	No	No

**Table A2:** Post-EnergiQ questionnaire results from the 38 active users.

Q4	Q5	Q6	Q7	Q8	Q9	Q10	Q11	Q12	Q13_a	Q13_b	Q13_c	Q13_d
5	4	5	5	5	4	4	4	4	1	1	1	1
5	5	5	5	5	5	5	4	4	0	1	1	1
5	5	4	5	5	5	4	4	5	1	1	0	1
4	4	5	5	5	5	5	5	5	1	1	1	1
5	5	5	3	5	5	5	5	5	1	1	1	1
5	5	5	4	5	5	5	4	5	1	1	1	1
4	5	5	5	4	5	5	5	5	1	1	1	0
4	5	5	5	5	5	5	4	4	1	1	1	1
5	5	4	4	5	4	5	4	5	1	1	1	0
4	5	4	5	5	5	5	5	4	1	1	0	0
5	5	5	5	5	4	5	4	5	1	1	1	1
5	5	4	4	5	5	5	5	5	1	1	0	0
5	5	5	5	4	5	5	5	5	1	1	1	1
5	5	5	4	4	5	5	5	4	1	1	0	1
5	4	5	5	5	5	5	5	5	1	1	1	1
5	5	4	4	5	5	5	5	5	1	1	1	1
5	5	4	5	5	5	4	5	5	1	1	0	1
5	5	4	5	4	5	5	5	5	1	1	0	1
4	5	4	5	4	4	4	5	4	1	1	0	1
5	5	4	5	5	5	5	5	5	0	1	1	1
4	5	5	3	5	5	5	4	5	0	1	1	1
5	4	4	4	5	5	4	5	4	1	1	1	1
4	5	5	4	4	5	5	5	5	1	1	1	1
5	5	5	5	5	5	5	5	5	1	1	1	1
5	5	5	5	5	4	5	5	5	0	0	0	1
4	5	5	3	4	4	5	5	4	1	1	1	1
5	5	5	5	4	5	4	4	5	1	1	1	1
4	4	4	5	5	5	5	4	5	1	1	0	0
5	5	5	5	4	5	5	4	5	1	1	0	1
4	4	4	4	5	5	5	5	5	1	1	1	1
4	5	5	5	4	4	5	5	5	1	1	0	1
4	5	4	4	4	4	5	5	4	1	1	1	1
5	5	5	3	5	4	5	5	4	1	1	1	1
4	4	5	5	4	5	5	5	4	1	1	1	1
4	5	5	3	5	4	5	4	4	0	0	1	1
4	4	5	5	4	5	4	5	5	1	1	1	1
5	5	4	3	5	5	5	4	4	1	1	1	1
5	5	4	3	4	5	4	5	5	1	1	1	1
